# A Brainstem reticulotegmental neural ensemble drives acoustic startle reflexes

**DOI:** 10.1038/s41467-021-26723-9

**Published:** 2021-11-04

**Authors:** Weiwei Guo, Sijia Fan, Dan Xiao, Hui Dong, Guangwei Xu, Zhikun Wan, Yuqian Ma, Zhen Wang, Tian Xue, Yifeng Zhou, Yulong Li, Wei Xiong

**Affiliations:** 1 Institute on Aging and Brain Disorders, The First Affiliated Hospital of USTC, Division of Life Sciences and Medicine, Hefei National Laboratory for Physical Sciences at the Microscale, University of Science and Technology of China, Hefei, 230026 China; 2 State Key Laboratory of Membrane Biology, Peking University School of Life Sciences, Beijing, 100871 China; 3 Shanghai Mental Health Center, Shanghai Jiao Tong University School of Medicine, Shanghai, P.R. China; 4 Center for Excellence in Brain Science and Intelligence Technology, Chinese Academy of Sciences, Shanghai, 200031 China; 5 PKU-IDG–McGovern Institute for Brain Research, Beijing, 100871 China

**Keywords:** Neural circuits, Reflexes

## Abstract

The reticulotegmental nucleus (RtTg) has long been recognized as a crucial component of brainstem reticular formation (RF). However, the function of RtTg and its related circuits remain elusive. Here, we report a role of the RtTg in startle reflex, a highly conserved innate defensive behaviour. Optogenetic activation of RtTg neurons evokes robust startle responses in mice. The glutamatergic neurons in the RtTg are significantly activated during acoustic startle reflexes (ASR). Chemogenetic inhibition of the RtTg glutamatergic neurons decreases the ASR amplitudes. Viral tracing reveals an ASR neural circuit that the cochlear nucleus carrying auditory information sends direct excitatory innervations to the RtTg glutamatergic neurons, which in turn project to spinal motor neurons. Together, our findings describe a functional role of RtTg and its related neural circuit in startle reflexes, and demonstrate how the RF connects auditory system with motor functions.

## Abstract (web summary)

The function of brainstem reticulotegmental nucleus (RtTg) and its related circuits are not fully understood. Here, the authors report a cochlear nucleus-RtTg-spinal motor neuron circuit that specifically mediates acoustic startle reflexes.

## Introduction

Located in the brainstem, the reticular formation (RF) has long been recognized as one of the most important part of the central nervous system (CNS). As its name suggested, the RF resembles a net-like structure composed of numerous ascending and descending nerve fibers along with various heterogeneous nuclei extending from the spinal cord to the thalamus^1^. This widespread projections allow the RF to act as a “command center” of the CNS. The midbrain RF together with its ascending tracts — also known as the reticular activating system (RAS) — project diffusely to the cerebellum, thalamus, cerebral cortex, and limbic system, functioning to promote the sleep-wake cycle, arousal, and consciousness^2,3^. Lesions to RAS result in impaired consciousness and even coma in some patients^4,5^. Descending fibers from the RF, mainly reticulospinal tracts, project to the spinal cord to coordinate respiratory control, pain perception, posture maintenance, and muscular reflexes^6–8^.

In mammals, the RF comprises over 100 individual nuclei. Most of the nuclei embedded in this structure have no precise cytoarchitectural boundaries^1^, rendering functional studies of these RF nuclei a challenging task. Situated at the ventral extreme of the pontine tegmentum, the reticulotegmental nucleus (RtTg) is one of the least investigated neural ensembles with clear delineations of territory in RF^9^. Early studies in primates indicated that RtTg might be involved with the oculomotor circuits crucial for smooth-pursuit eye movement^10^. On the other hand, controversial conclusions have been demonstrated regarding to the functional relationship between RtTg and locomotion. Unit recordings in monkeys and cats have revealed increased RtTg neuronal activities during limb movements^11,12^. Another early study in mice also observed consistent activation of RtTg during motor seizure^13^. Still, other researchers have observed increased forward locomotion in rats following electronic lesion of the RtTg^14^.

Although sporadic studies on RtTg have been reported over the past few decades, the precise functional role of the RtTg and its brain-wide upstream and downstream projections have not been investigated thoroughly. Recently, the development of cell-type-specific viral tracing approaches as well as optogenetic/chemogenetic tools has enabled more accurate discovery and elucidation of the potential roles and circuit connections in a target brain region^15^. Using these cutting-edge techniques combined with in vitro/in vivo electrophysiological recordings, we surprisingly found that the RtTg controls a conservative startle response, and identified a neural circuit from the cochlear nucleus (CN) to spinal motor neurons that underlies this innate defensive behaviour.

## Results

### Optogenetic activation of RtTg neurons elicits startle reflexes

**Fig. 1:**
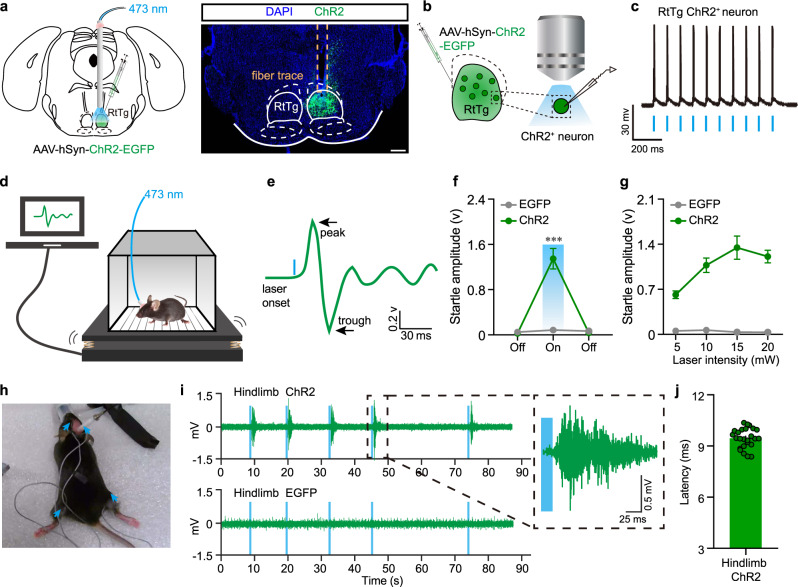
Optogenetic activation of RtTg leads to stereotypical startle behaviour. **a** Left: schematic for optogenetic activation of the RtTg. Right: a representative image confirming ChR2-EGFP expression in the RtTg, and the optical fiber trace. Scale bar, 400 μm. **b** Schematic for the optogenetic activation and the simultaneous whole-cell patch-clamp recording of RtTg EGFP^+^ neurons in the RtTg brain slice of mice receiving an intra-RtTg injection of the AAV-hSyn-ChR2-EGFP. **c** Sample traces showing the photostimulation (blue bars, 1 ms)-evoked action potentials in RtTg EGFP^+^ neurons. **d** Schematic for the optogenetic activation of the RtTg and simultaneous detection of startle reflexes. **e** Startle amplitude was defined as the largest peak-to-peak response occurred within 200 ms after the onset of the laser stimulus. **f** Startle amplitudes evoked by opto-stimulation (blue bars) of the RtTg neurons expressing ChR2 or EGFP (*n* = 15 mice per group, two-sided unpaired *t-tests, P* = 5.78 × 10^−6^). **g** Startle amplitudes at different laser intensities in the ChR2 and EGFP group (*n* = 15 mice per group). **h** Representative image showing the experimental setup for EMG recording during optogenetic activation of RtTg neurons. Blue arrows indicate positions for electrode insertion. **i** Example of the entire and locally amplified EMG traces showing activities of mouse hindlimb muscles during optogenetic activation (blue bar) of RtTg neurons. **j** Quantification of latency for hindlimb muscle EMG activities after optogenetic activation of the RtTg (*n* = 5 mice, 25 bouts in total). Error bar represent mean ± s.e.m. ***P < 0.001.

We first conducted optogenetic experiments^16^ to examine the behavioural readout for RtTg neuronal activation. We unilaterally injected adeno-associated viruses (AAV) expressing Channelrhodopsin-2 (AAV-hSyn-ChR2-EGFP) into the RtTg of mice, followed by implantation of an optical fiber above the injection site (Fig. 1a). Mice transfected with AAV-hSyn-EGFP were used as controls. Brain slice patch-clamp recordings showed that a light-pulse train triggered action potentials in the RtTg ChR2^+^ neurons, indicating the effectiveness of RtTg neuron activation (Fig. 1b, c). Strikingly, optogenetic activation of RtTg neurons reliably evoked robust startle reflexes, which were defined as involuntarily innate defensive response occurring rapidly (often in milliseconds) after the onset of aversive stimuli (Fig. 1d–f and Supplementary Movie 1). Moreover, the startle amplitudes were positively correlated to the laser intensities (Fig. 1g). As a control, no startle reflex was observed upon photostimulation of RtTg neurons expressing EGFP only (Fig. 1f, g and Supplementary Movie 2). It is worth noting that the optogenetic manipulation was strictly limited to the RtTg region without influencing the adjacent caudal pontine reticular nucleus (PnC) (Supplementary Fig. 1), which has been already recognized to be critical for startle reflex by previous studies^17–19^.

Previous reports demonstrated that through all mammals, the stereotypical startle pattern consists of a fast contraction of neck, facial and skeletal muscles^17^. We therefore examined the electromyographic (EMG) activities in the neck and hindlimb of mice during optogenetic activation of RtTg neurons. As expected, we consistently detected robust EMG activities in both neck and hindlimb muscles following every single optogenetic stimulation pulse (Fig. 1h, i and Supplementary Fig. 2a, b). The EMG activities occurred very shortly after optogenetic activation of RtTg neurons, with a mean latency of 5.75 ± 0.38 ms for neck muscle (Supplementary Fig. 2c) and 9.43 ± 0.12 ms for hindlimb muscle (Fig. 1j) respectively.

### RtTg inactivation specifically suppresses acoustic startle reflexes

**Fig. 2:**
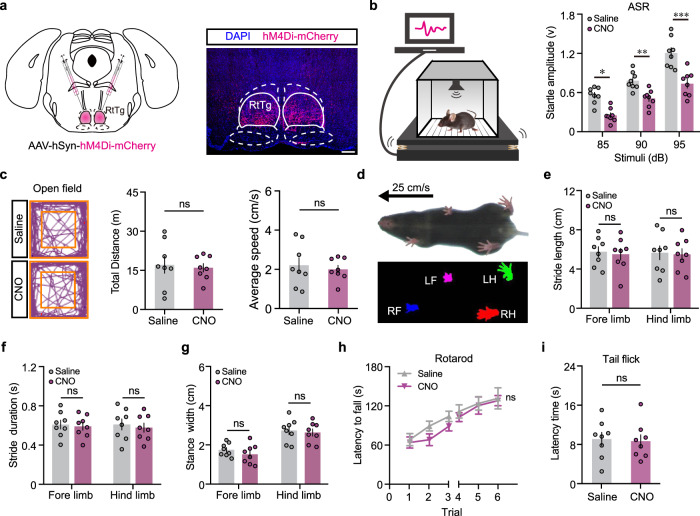
Behavioural consequences of RtTg inactivation. **a** Left: schematic for chemogenetic inactivation of RtTg neurons. Right: a representative image confirming hM4Di-mCherry expression. Scale bar, 300 μm. **b** Left: schematic for the ASR paradigm. Right: quantitative graph showing the effect of RtTg inactivation on ASR amplitudes (*n* = 8 per group; *t* = 2.417, *P* = 0.02 (85 dB); *t* = 3.429, *P* = 0.0014 (90 dB); *t* = 5.976, *P* = 4.3 × 10^−7^ (95 dB)). **c** Left: representative movement tracks of hM4Di-infected mice after i.p. injection of saline or CNO. Quantification of total distances (middle, *n* = 8 per group; *P* = 0.7637) and average speed (right, *n* = 8 per group; *P* = 0.6411) during open field test. **d** Representative images showing a mouse walking on treadmill and paw prints detected from a video settled under the treadmill. **e**–**g** Digi Gait analysis for hM4Di infected mice after i.p. injection of saline or CNO (*n* = 8 per group; for e: *t* = 0.2945, *P* = 0.7705 (fore limb); *t* = 0.1914, *P* = 0.8496 (hindlimb); for f: *t* = 0.2689, *P* = 0.7899 (fore limb); *t* = 0.4781, *P* = 0.6363 (hindlimb); for g: *t* = 0.9117, *P* = 0.3697 (fore limb); *t* = 0.4126, *P* = 0.6831 (hindlimb)). **h** Latency to fall on accelerating rotarod of hM4Di-infected mice after i.p. injection of saline or CNO (*n* = 8 per group; *F* = 0.2, *P* = 0.9346). **i** Tail-flick latency of hM4Di-infected mice after i.p. injection of saline or CNO (*n* = 8 per group, *P* = 0.8269). Error bar represent mean ± s.e.m. Significance was assessed using two-sided unpaired *t-*tests in c,i, and two-way ANOVA combining with FDR corrections in **b**, **e**–**h**. * *P* < 0.05; ** *P* < 0.01; *** *P* < 0.001; ns, not significant.

We next used chemogenetic suppression-based loss-of-function experiments to further verify the potential role of RtTg in acoustic startle reflex (ASR), a major form of startle behaviours. Specifically, the AAV virus expressing mCherry-tagged hM4Di^20^ — an inhibitory designer receptor exclusively activated by the designer drug (DREADD) — was bilaterally injected into the RtTg (Fig. 2a). We then employed a well-established paradigm^21^ to trigger and measure the ASR in mice. Transient suppression of RtTg neurons by intraperitoneal (i.p.) injection of clozapine-N-oxide (CNO) significantly decreased ASR amplitudes (Fig. 2b) without affecting other innate behaviours including motor coordination (Fig. 2c–h) and pain perception (Fig. 2i).

We then examined the effects of Caspase3-based ablation^22^ of RtTg neurons on ASR. Specifically, a mixture of AAV-hSyn-Cre and AAV-flex-taCasp3-TEVp was bilaterally co-injected into the RtTg (Supplementary Fig. 3a). Immunohistochemical analysis showed a remarkable reduction in the number of cells stained positive for neuronal nuclear antigen (NeuN^+^) in the targeted RtTg region, confirming efficient ablation (Supplementary Fig. 3b–f). Compared with the control group, mice with RtTg lesions exhibited significantly decreased ASR amplitude (Supplementary Fig. 3g). In contrast, the RtTg ablation did not affect motor coordination (Supplementary Fig. 3h–m), arousal (Supplementary Fig. 3n), and pain perception (Supplementary Fig. 3o). Together, these results suggest that the RtTg plays an essential role specifically in startle behaviours.

### The activities of RtTg glutamatergic neurons augment during ASR

**Fig. 3:**
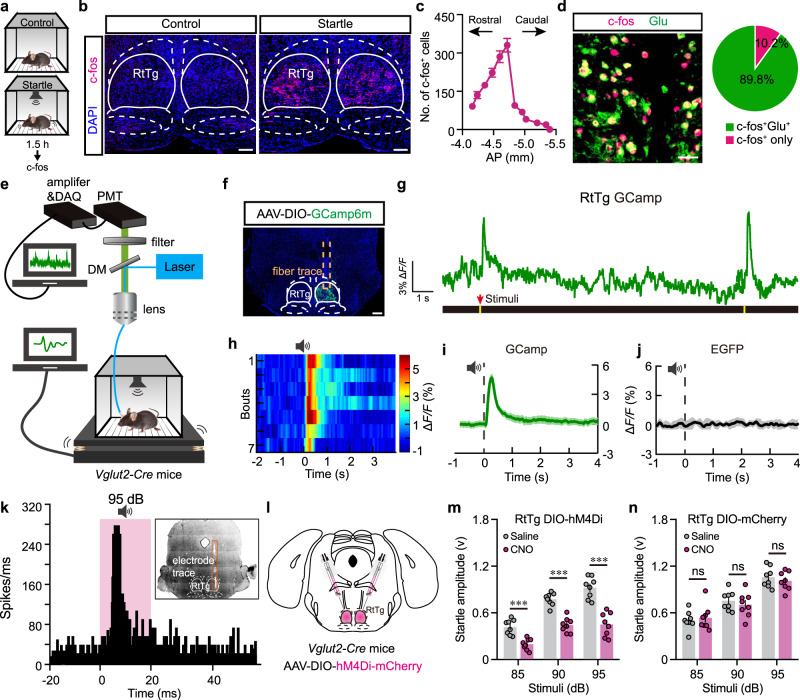
RtTg glutamatergic neurons mediates ASR. **a** Schematic of ASR c-fos experiment. **b** Representative images showing control and 95 dB acoustic stimuli-induced c-fos expression in the RtTg. Scale bar, 200 μm. **c** Quantification of the c-fos^+^ cell number in consecutive RtTg sections (*n* = 6 mice). AP, distance anterior or posterior to Bregma. **d** Left: a representative image showing RtTg c-fos^+^ cells co-labelled with glutamate. Scale bar, 50 μm. Right: pie chart indicating the percentage of c-fos^+^ neurons co-labelled with or without glutamate. **e** Schematic of fiber photometry setup. **f** A representative image confirming GCamp6m expression in the RtTg. Scale bar, 400 μm. **g**, **h** Representative traces (g) and heat map (h) of calcium signal changes of RtTg glutamatergic neurons aligned to the acoustic stimuli onset. **i**, **j** Quantifications of calcium (**i**, *n* = 35 bouts from 5 mice) and EGFP (**j**, *n* = 25 bouts from 5 mice) signal changes of RtTg glutamatergic neurons aligned to the acoustic stimuli onset. The thick line indicates the mean and the area shaded in lighter color indicates s.e.m. **k** The average peristimulus time histogram showing RtTg neuronal activities evoked by acoustic stimulus. **l** Schematic for chemogenetic inhibition of glutamatergic neurons in the RtTg of *Vglut2-Cre* mice. **m**, **n** ASR amplitudes of *Vglut2-Cre* mice receiving bilateral injection of AAV-DIO-hM4Di-mCherry (**m**) or control AAV-DIO-mCherry (**n**) into the RtTg, followed by i.p. administration of CNO or saline (n = 8 per group; for m: *t* = 4.026, *P* = 0.0002 (85 dB); *t* = 6.049, *P* = 3.38 × 10^−7^ (90 dB); *t* = 8.626, *P* = 7.63 × 10^−11^ (95 dB); for *n*: *t* = 0.4851, *P* = 0.6301 (85 dB); *t* = 0.7525, *P* = 0.4559 (90 dB); *t* = 0.6869, *P* = 0.4959 (95 dB)). All data are presented as the mean ± s.e.m. Significance was assessed using two-way ANOVA combining with FDR corrections. *** *P* < 0.001; ns, not significant.

We next examined the expression of c-fos, a marker for recent neural activity^23^, in the RtTg of mice exposed to 95 dB acoustic stimuli. In addition to several brain nuclei with well-characterized functional involvement in the central auditory system — including the CN and the auditory cortex (AuC)^24^ — we also observed robust acoustic-stimuli-induced activation of RtTg neurons (Supplementary Fig. 4 and Fig. 3a, b). Pursuing this, we generated a clear map of the c-fos expression pattern of RtTg during ASR by collecting all consecutive coronal sections covering the entire RtTg area. Quantification analysis revealed a greater density of c-fos^+^ cells in the rostral and medial parts of the RtTg (Fig. 3c and Supplementary Fig. 5). A non-acoustic stimulus such as air puff can also elicit startle reflexes in rodents^25^. However, we did not detect any changed c-fos expression in the RtTg of mice following air puff-induced startle reflexes (Supplementary Fig. 6).

To further examine whether RtTg neurons are selectively responsive to strong stimuli that drive the startle or just generally sensitive to auditory cues, we performed RtTg c-fos staining after mice were introduced to white noise stimuli with weaker intensities (45, 50, 55, 60 dB). These weak stimuli have been shown to be audible in mice^26^, but were insufficient to elicit startle reflex (Supplementary Fig. 7). As shown in Supplementary Fig. 8a, the c-fos expression level of RtTg under 45–60 dB noise stimuli was extremely low and had no significant correlation with these weak white noise intensities. In comparison, the RtTg total c-fos^+^ cell number was well correlated with startle-eliciting white noise intensities (Supplementary Fig. 8b) as well as startle amplitudes (Supplementary Fig. 8c). These results demonstrate that the activity of RtTg indeed correlates with the startle response.

Screening via the Allen Brain Database indicated that RtTg neurons feature a high expression of vesicular glutamate transporter 2 (*VGLUT2*) mRNA, a classic marker for excitatory glutamatergic neurons. Consistently, our immunostaining results revealed that nearly 90% of c-fos^+^ RtTg neurons co-labelled with glutamate (Fig. 3d), confirming the excitatory nature of these ASR-activated RtTg neurons.

To further monitor the dynamics of RtTg glutamatergic neurons during ASR, we injected a genetically encoded Cre-dependent calcium indicator (AAV-DIO-Ef1α-GCamp6m) into the RtTg of *Vglut2-Cre* mice^27^ and performed fiber photometry^28^ (Fig. 3e). Mice transfected with AAV-DIO-Ef1α-EGFP were used as controls. Fluorescent signal fluctuations were recorded simultaneously with ASR data. As with our initial observations based on c-fos experiments, there were significantly elevated calcium signals in RtTg glutamatergic neurons upon the onset of 95 dB stimuli (Fig. 3f–i), whereas no signal changes were detected in control mice expressing EGFP (Supplementary Fig. 9 and Fig. 3j). The changes in calcium signals of RtTg glutamatergic neurons exhibited a positive correlation with the acoustic stimulus intensities (Supplementary Fig. 10). In contrast, we did not detect increased calcium signals of RtTg glutamatergic neurons after the onset of air puff stimulations (Supplementary Fig. 11).

Given that startle reflex occurs rapidly in response to abrupt aversive stimuli, we next performed in vivo recording of RtTg neurons during ASR to determine whether RtTg neurons also response with short latency compatible with the initiation of the startle reflex. Mice were introduced to white noise stimuli followed by sampling the RtTg neuronal firing rate of multiple units. RtTg neuronal activity increased significantly upon the onset of acoustic stimulus with a mean spike latency of 5.12 ± 0.47 ms (Fig. 3k). Furthermore, we observed a gradual increase in firing rates with elevated stimulus intensities (Supplementary Fig. 12). These results indicate that RtTg neurons can quickly response to acoustic stimuli.

### Chemogenetic inhibition of RtTg glutamatergic neurons suppresses ASR

We further examined whether chemogenetic inhibition of RtTg glutamatergic neurons could reduce ASR amplitudes. A Cre-dependent AAV expressing mCherry-tagged hM4Di was bilaterally injected into the RtTg of *Vglut2-Cre* mice (Fig. 3l and Supplementary Fig. 13). In acute RtTg brain slices, bath application of CNO reduced the membrane potential as well as the firing frequency of hM4Di^+^ RtTg neurons, confirming the efficacy of hM4Di-mediated inhibition (Supplementary Fig. 14). Furthermore, i.p. injected CNO significantly decreased ASR amplitudes (Fig. 3m). Note that these effects cannot be directly attributed to CNO or its metabolites, as the mCherry control mice injected with CNO exhibited no such decrease in startle response (Fig. 3n). Taken together, these results indicate that the activity of RtTg glutamatergic neurons is capable of, and necessary for driving startle reflexes.

### Chemogenetic inhibition of RtTg GABAergic neurons enhances ASR

According to the Allen Brain database, neurons in the central region of the RtTg are predominantly glutamatergic, whereas neurons expressing high levels of mRNA transcripts for inhibitory GABAergic neuron marker, vesicular GABA transporter (*VGAT*), were mainly distributed in the pericentral region of the RtTg (Supplementary Fig. 15a). Consistently, in this study, immunostaining of the brain sections from *GAD1-EGFP* transgenic mice^29^ further demonstrated the shell-distribution pattern of the inhibitory neurons embracing glutamatergic neurons in the central RtTg (Supplementary Fig. 15b). Moreover, chemogenetic inhibition of the pericentral RtTg GABAergic neurons in *Vgat-Cre* mice resulted in significantly enhanced ASR amplitudes, suggesting a negative regulatory role of RtTg GABAergic neurons in startle reflexes (Supplementary Fig. 15c, d).

### RtTg receives direct projections from CN

**Fig. 4:**
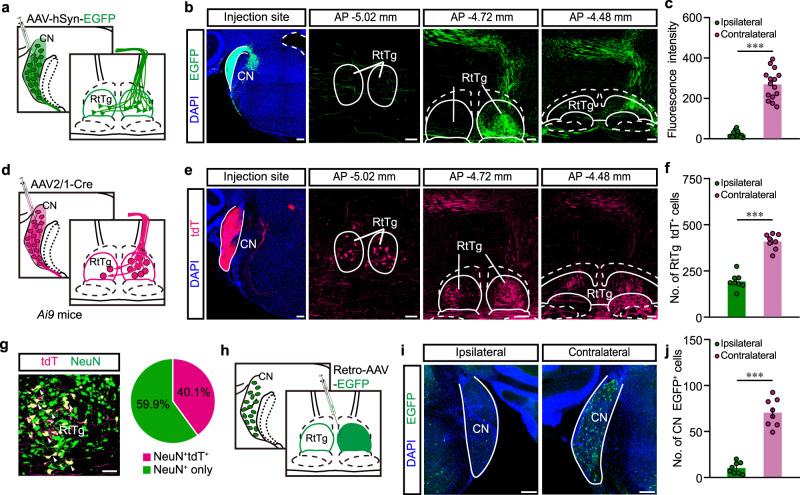
Identification of a CN-RtTg direct projection. **a** Schematic for the anterograde tracing from CN to RtTg. **b** Representative images showing CN injection site and EGFP^+^ presynaptic terminals in RtTg. Scale bar, 200 μm. **c** Quantitative fluorescence intensity of CN EGFP^+^ presynaptic terminals in the ipsilateral and contralateral RtTg (*n* = 15; *P* = 8.87 × 10^−9^). **d** Schematic for the anterograde transsynaptic tracing from CN to RtTg. **e** Representative images showing CN injection site and the tdT^+^ cells in RtTg. Scale bar, 200 μm. **f** Quantification of tdT^+^ cell numbers in the ipsilateral and contralateral RtTg (*n* = 8; *P* = 3.8 × 10^−7^). **g** Left: a representative image showing tdT^+^ RtTg neurons co-labelled with NeuN (white arrowheads). Scale bar, 50 μm. Right: pie chart indicating the percentage of NeuN^+^ neurons co-labelled with or without tdT. **h** Schematic for the retrograde tracing from RtTg to CN. **i** Representative images showing EGFP^+^ neurons in ipsilateral and contralateral CN. Scale bar, 200 μm. **j** Quantification of EGFP^+^ cells number in the ipsilateral and contralateral CN (*n* = 8; *P* = 5.09 × 10^−6^). Error bar represents mean ± s.e.m. Significance was assessed using two-sided paired *t-*tests in **c**, **f**, **j**. ***P < 0.001.

Given that the CN is the initial central auditory structure to receive input from the cochlear via the auditory nerve^30^, our discovery that the RtTg mediates ASR motivated us to examine if the RtTg is a downstream target of the CN. We conducted projection tracing experiments, wherein AAV-hSyn-EGFP^31^ was unilaterally injected into the CN (Fig. 4a). In addition to brain regions such as the lateral superior olivary nuclei (LSO) and the inferior colliculus (IC) known to receive direct CN inputs^24,32^ (Supplementary Fig. 16a–c), confocal images of serial coronal sections revealed dense EGFP^+^ terminals in the RtTg (Fig. 4b and Supplementary Fig. 17). Indicating a strong trend for cross-hemispherical projection, we noted that the EGFP signal was much stronger in contralateral RtTg than ipsilateral RtTg (Fig. 4c).

Next, we unilaterally injected the anterograde transsynaptic tracer AAV2/1-hSyn-Cre^33^ into the CN of *Ai9* mice, a Cre-dependent tdTomato (tdT) reporter line^34^ (Fig. 4d). Consistent with our results from the aforementioned experiments with the AAV-hSyn-EGFP (Supplementary Fig. 16d, e), we detected abundant tdT^+^ cells in RtTg, with especially dense labelling of neuron populations in the contralateral RtTg (Fig. 4e, f and Supplementary Fig. 18). Examining this RtTg population specifically, we noted that 40.1% (NeuN^+^tdT^+^ cells) of total RtTg neurons received CN innervation (Fig. 4g and Supplementary Fig. 19).

To further confirm the existence of CN-RtTg projections, we unilaterally injected Retro-AAV-EGFP^35^ into the RtTg (Fig. 4h, Supplementary Fig. 20). Abundant EGFP^+^ cells were observed in the CN, with EGFP^+^ cells number much higher in the contralateral CN than that in the ipsilateral CN (Fig. 4i, j). Together, these results demonstrate that the CN directly innervates the RtTg.

### The glutamatergic CN-RtTg projections mediate startle reflexes

**Fig. 5:**
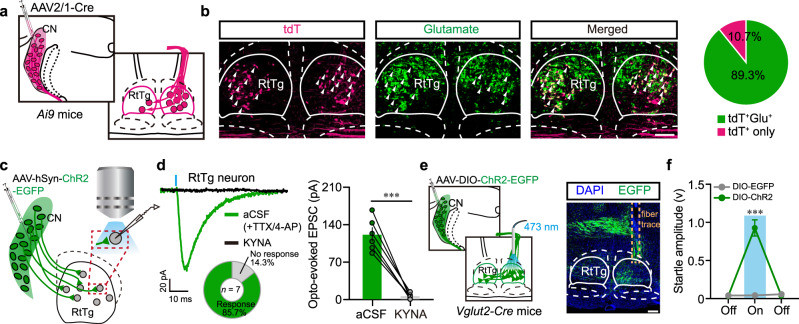
The CN sends monosynaptic glutamatergic projections to RtTg glutamatergic neurons. **a** Schematic for the anterograde tracing from CN to RtTg. **b** Left: representative images showing RtTg tdT^+^ neurons co-labelled with glutamate (white arrowheads). Scale bar, 300 μm. Right: pie chart indicating the percentage of RtTg tdT^+^ neurons co-labelled with or without glutamate. **c** Schematic for the optogenetic activation of CN neuronal terminals and the simultaneous whole-cell patch clamp recording of RtTg neurons. **d** Sample traces (left) and quantitative analysis (right) for EPSCs of RtTg neurons evoked by photostimulation (blue bar, 1 ms) before and after bath application of KYNA. TTX and 4-AP were added into aCSF (*n* = 6 cells from 4 mice; *P* = 0.0003). The doughnut chart shows the percentage of RtTg neurons responsive or not responsive to the photostimulation. **e** Left: schematic for the optogenetic activation of CN glutamatergic neuron terminals in the RtTg. Right: a representative image showing EGFP^+^ terminals in the RtTg. Scale bar, 300 μm. **f** Startle amplitudes evoked by photostimulation of ChR2^+^ terminals or EGFP^+^ terminals in the RtTg of *Vglut2-Cre* mice (*n* = 15 stimuli from 5 mice per group; *P* = 7.76 × 10^−7^). Error bar represents mean ± s.e.m. Significance was assessed using two-sided paired *t-tests* in **d**, and two-sided unpaired *t-tests* in **f**. *** *P* < 0.001; ns, not significant.

To further investigate the nature of CN-RtTg neural projections, we first tested whether the CN innervate RtTg glutamatergic neurons, which has been proved to mediate startle reflexes. Anterograde transsynaptic tracer AAV2/1-hSyn-Cre was unilaterally injected into the CN of *Ai9* mice (Fig. 5a), and consecutive RtTg coronal sections were collected for glutamate immunostaining. As expected, nearly 90% of tdT^+^ RtTg neurons contain glutamate, suggesting that CN neurons project extensively to RtTg glutamatergic neurons (Fig. 5b and Supplementary Fig. 21).

We next examined the type of neurotransmission between CN and RtTg by performing whole-cell patch-clamp recordings of the RtTg brain slices prepared from mice that received virally-encoded ChR2 injection into their CN (Fig. 5c). Photostimulation of CN ChR2^+^ terminals evoked excitatory postsynaptic currents (EPSCs) in most (6 of 7) RtTg neurons treated with Tetrodotoxin (TTX) and 4-aminopyridine (4-AP)^36^. Bath application of the glutamate receptor antagonist kynurenic acid (KYNA) completely blocked the light-evoked EPSCs (Fig. 5d). Collectively, these results demonstrate that CN neurons form monosynaptic glutamatergic synapses with RtTg neurons.

We then employed projection-based optogenetic-activation and chemogenetic-inactivation approaches to explore the functional contribution of this excitatory CN-RtTg innervation in freely moving mice. A Cre-dependent ChR2 or hM4Di virus was unilaterally/bilaterally injected into the CN of *Vglut2-Cre* mice, followed by implantation of an optical fiber or a guide cannula above the RtTg (Fig. 5e and Supplementary Fig. 22a, b). As expected, optogenetic activation of ChR2 terminals, but not EGFP terminals, in the RtTg elicited robust startle reflexes (Fig. 5f). On the contrary, chemogenetic inhibition of hM4Di terminals in the RtTg dramatically decreased ASR amplitudes (Supplementary Fig. 22c). These results further support that the CN-RtTg glutamatergic projections mediate ASR.

### Spinal motor neurons are downstream targets of the RtTg

**Fig. 6:**
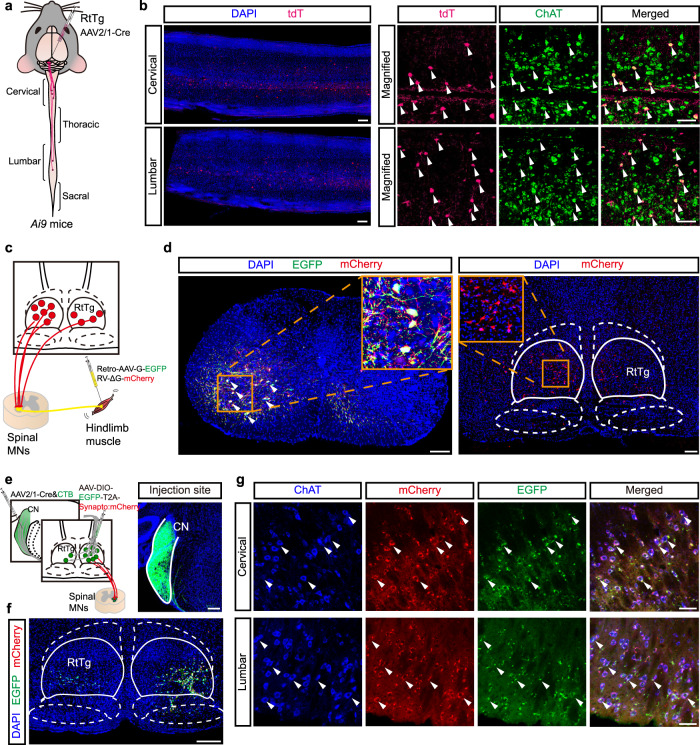
Viral tracing of the CN-RtTg-MNs circuit. **a** Schematic for the AAV2/1-Cre-based anterograde transsynaptic tracing from the RtTg to multiple spinal cord segments. **b** Representative images showing the tdT^+^ neuron labelling in transverse sections of spinal cervical and lumbar segments, and the co-labelling of tdT with ChAT (white arrowheads). Scale bar, 100 μm. **c** Schematic for rabies virus-based retrograde monosynaptic tracing from hindlimb muscles to the RtTg. **d** Representative images showing EGFP^+^mCherry^+^ spinal starter MNs (left) and mCherry^+^ neurons in the RtTg (right). Scale bar, 200 μm. **e** Left: schematic for the multilevel anterograde tracing from the CN to the spinal cord by injecting AAV2/1-Cre (mixed with CTB to visualize injection site) into the CN, followed by the injection of AAV-DIO-EGFP-T2A-Synaptophysin:mCherry into the contralateral RtTg. Right: a representative image showing CN injection site. Scale bar, 200 μm. **f** A representative image showing Cre-dependent expression of EGFP in RtTg neuron cytosol. Scale bar, 300 μm. **g** Representative images showing co-existence (white arrowheads) of the mCherry^+^ presynaptic puncta of RtTg neurons and the spinal cervical segment ChAT^+^ MNs. Scale bar, 200 μm (Cervical, Lumbar) and 5  μm (Merged).

Given the above data indicating that optogenetic activation of the RtTg instantly evoked EMG activities in hindlimb muscles, we questioned whether the RtTg neurons specifically project to spinal motor neurons (MNs), as these neurons are known to mediate muscular movement. To define the segments of the spinal cord where RtTg neurons may terminate, we unilaterally injected the anterograde transsynaptic tracer AAV2/1-hSyn-Cre into the RtTg of *Ai9* mice, followed by imaging of the spinal cord transverse sections from cervical to sacral segments (Fig. 6a). We observed that tdT^+^ neurons are predominantly distributed in cervical and lumbar spinal cord segments, which have been previously demonstrated to harbor most of the neurons that innervate the limb muscles^37,38^. Immunostaining showed that these tdT^+^ neurons were co-labelled with an MN-specific marker, choline acetyltransferase (ChAT) (Fig. 6b and Supplementary Fig. 23, 24).

Given previous studies reporting that some spinal interneurons also express ChAT^39^, to confirm that the RtTg indeed innervates muscle-controlling spinal MNs, we next performed rabies virus-based retrograde mono-transsynaptic tracing experiments^40^. Specifically, Retro-AAV encoding G protein and EGFP was injected into the hindlimb muscles of mice, followed by the injection of RV-ΔG-mCherry 3 weeks later (Fig. 6c). Using this approach we could specifically label the muscle-projecting spinal MNs. Starter MNs co-expressing EGFP and mCherry were found in the ventral horn of the spinal lumber segment (Fig. 6d). Furthermore, trans-synaptically labelled premotor neurons (expressing mCherry only) were abundantly found in the RtTg (Fig. 6d). These results indicate the existence of monosynaptic connections between RtTg and spinal muscle-controlling MNs.

To further examine whether the MN-projecting RtTg neurons also receive CN innervation, we unilaterally injected AAV2/1-hSyn-Cre into the CN and AAV-DIO-EGFP-T2A-Synaptophysin:mCherry^41^ into the contralateral RtTg (Fig. 6e). After confirming that transsynaptic spreading of Cre successfully labelled the RtTg neuron cytosol (EGFP) (Fig. 6f), we observed that the mCherry^+^ presynaptic terminals of these RtTg neurons closely contact ChAT^+^ spinal MNs in the spinal cervical and lumbar segments (Fig. 6g). Thus, we demonstrated a brainstem reticulotegmental neural ensemble that connects the auditory system with the motor functions, which mediates startle reflexes.

## Discussion

Although sporadic reports from human and animal studies have revealed involvement of RtTg in smooth-pursuit eye movement as well as limb movements^10–14^, the research about RtTg is still very limited. In this study, we demonstrate that the RtTg acts as a startle-controlling nucleus. This conclusion is strongly supported by sufficient data from the present study: (1) Optogenetic activation of RtTg neurons elicited robust startle reflexes; (2) Chemogenetic inactivation and viral ablation of RtTg neurons suppressed startle amplitudes; (3) RtTg neurons exhibited increased calcium activities and firing spikes during ASR; (4) Activation of the RtTg induced EMG activities of neck and hindlimb muscles within millisecond latency, consistent with the nature of startle reflexes, a quick alert to sudden aversive stimuli.

Numerous early studies demonstrated that the PnC nucleus might play a critical role in startle reflexes^17–19,42,43^. According to the mouse brain atlas^44^, the RtTg situates closely adjacent to the PnC. Therefore, in this study, when manipulating the RtTg such as Casp3-based lesion, optogenetic activation as well as chemogenetic inhibition, we always checked whether there was any collateral influence on the PnC neurons, and any data with cross-contamination of the PnC were eliminated from the analysis. In other words, the identification of functional roles of the RtTg in the startle reflex should not be artifacts caused by destruction or stimulation of the adjacent PnC nucleus. Thus, based on previous and our studies, both the PnC and the RtTg are critically involved in mediating the startle reflex. In fact, the phenomenon that two different brain nuclei mediate the same behaviour does exists^45,46^. It is worth noting that in most previous studies, PnC neurons were activated by strong acoustic stimulus (≥110 dB)^47–49^, while in the present study, we used relatively lower acoustic stimulus (75–95 dB) to trigger the startle reflex and also observed the activation of RtTg neurons. Thus, it is possible that the PnC and the RtTg may have different sensitivities to acoustic stimulus patterns, such as intensity, frequency, duration, and sound type. Further investigation needs to be done to explore these possibilities and the potential structural as well as a functional connection between the RtTg and the PnC.

Although innate, startle reflexes can be modulated by fear emotional state, known as fear-potentiated startle (FPS)^50^. The abnormally exaggerated startle reflex is a common feature across different fear-associated neuropsychiatric disorders including post-traumatic stress disorder, panic disorder, and specific phobia^50–52^. To date, however, circuit mechanisms for FPS remain unclear, and the therapeutic treatments to relieve this symptom are obscure. Previous studies indicate that the amygdala-PnC neural projections may engage in the modulation of the startle reflex by fear emotions. As a newly identified startle-controlling nucleus, the RtTg may also receive projections from fear-encoding upstream nuclei and contribute to the development of FPS. For instance, in the present study, when performing retrograde viral tracing from the RtTg, we did observe several fear-related upstream nuclei, such as the dorsal raphe nucleus and the lateral habenular nucleus (Supplementary Fig. 20). Thus, dissection of the circuit basis of RtTg-mediated startle reflex should provide insight for further study of the mechanism underlying abnormal exaggerated startle symptoms observed in many psychiatric disorders.

## Methods

### Animals

All animal experiments were conducted following protocols approved by the Institutional Animal Care and Use Committee of the University of Science and Technology of China (USTC). C57BL/6 J, *Vglut2-Cre*, *Vgat-Cre,* and *GAD1-EGFP* mice were purchased from Beijing Vital River Laboratory or Jackson Laboratories. Mice were group-housed (3-5 animals per cage) in a stable environment (23-25 °C ambient temperature and 50% humidity). Mice were maintained in a 12-hour light/dark cycle (light on at 7 a.m.) with free access to ad libitum chow food and water, and were allocated randomly to control and experimental groups. All experiments were performed on 8-week male mice to avoid potential sex as well as age differences. All experimental data were obtained by individuals blind to the experimental condition.

### Acoustic startle reflex (ASR) test

After a 30 min period of acclimation to the testing room, mice were placed in a chamber within the darkened startle apparatus (MED Associate) for the ASR test. Broadband white noise were emitted from a high-frequency speaker mounted above the apparatus. A load cell platform under the chamber digitize the pressure generated by startled mice, and ASR amplitude was analyzed using Startle Reflex Software (MED Associate). After 5 min habituation period inside the startle chamber, mice received 30 startle trials (20-ms white noise stimuli with intensities of 85, 90, or 95 dB). Each of the three white noise stimuli was applied 10 times in random order at random inter-trial intervals (ITIs) ranging from 58-63 s. The resulting startle reflexes of the mice in the startle chamber were recorded during 600 ms after acoustic stimuli onset. ASR amplitude of each trial was defined as the largest peak-to-trough response that occurred within 200 ms after the onset of the startle stimulus. ASR amplitude of each stimulus intensity was calculated as the mean amplitude of 10 startle trials.

### Open field test

After a 30 min period of acclimation to the testing room, mice were individually placed into the open field area (45 × 45 cm^2^) and their spontaneous motor activities were measured for 10 min. The area was cleaned using 75% ethanol between each trial. Total distance together with time spent in the center zone were quantified automatically using ANY-maze system (Global Biotech Inc.).

### Rotarod test

Before the training session started, mice were placed on the stationary rod for 5-min acclimation. Mice were trained three sessions per day for 2 days, with a 30 min inter-session interval. In each training session, mice were placed on the rotating rod accelerated from 4 rpm to 60 rpm in 300 s. Total time was recorded (XR1514, Xinruan) until the mice fell off from the rod or gripped and circled around two times. Mice staying more than 300 s were taken manually from the rod, with total time recorded as 300 s. After finishing the session, the apparatus was cleaned using 75% ethanol.

### Tail-flick test

Tail-flick latency was measured using a tail-flick test apparatus (Ugo Basile). For each test, a radiant light beam was focused on a ventral portion of the mouse’s tail (1.5–2 cm from its top). Light exposure was ended automatically once the mouse exhibit tail-flick motion. A 30 s cut-off time was employed to avoid skin injury. At the end of each test, the apparatus was cleaned with 75% ethanol alcohol.

### Gait analysis

Gait analysis was conducted using the DigiGait imaging system (Mouse Specifics Inc.). Mice were placed individually on a transparent treadmill enclosed by a plexiglass compartment and have 3–5 min to freely explore the compartment while the treadmill remained still. After brief acclimation, the belt was started at an initial speed of 10 cm/s and gradually increased to a constant speed of 25 cm/s. Digital videos of paw movement were acquired using a camera mounted under the treadmill. For each mouse, approximately 5 s of video were selected based on continuous movement. Videos were then analyzed using DigiGait software to automatically calculate gait parameters including stride-related variables as well as stance width.

### Pupil monitoring

Mice were surgically prepared for head-fixed monitoring of pupil diameter. After a 3-day recovery period, mice were placed individually in a custom-made restrainer and were aroused using air-puff stimuli generated by a nitrogen tank connected to a solenoid-operated piston pump at random ITIs ranging from 60–80 s. A small tube was placed behind the mice’s head to deliver air puff. Pupil sizes were recorded using an infrared camera-based eye-tracking system (ISCAN systems, USA), filtered (CyberAmp, USA), and digitized (CED, UK) for offline analysis. %changes of pupil diameter during air puff were calculated using custom-made MATLAB routines (The MathWorks, Natick, MA, USA).

### Stereotaxic injection

Mice were anesthetized with an intraperitoneal injection of pentobarbital sodium (0.5%, 0.01 ml/g) and mounted on a stereotaxic apparatus (68015, RWD Life Science). Ophthalmic ointment was applied to prevent the eyes from drying. The skull was exposed by a midline scalp incision, and a craniotomy was drilled unilaterally or bilaterally above each cannula or optical fiber implantation or injection site. Viruses were delivered at a rate of 50 nl/min using glass pipettes connected to a pump (Legato130, KD Scientific). After completion of the injection, the glass pipettes were held in place for 10 min before being withdrawn to allow for diffusion of viruses. The implanted cannula or optical fiber was stabilized to the skull with dental cement. The animals were allowed to recover from anesthesia on a heating pad and housed for 2-4 weeks after surgery. Coordinates used for RtTg injection were as followed: bregma −3.85 mm, lateral ± 0.47 mm, and dura −4.75 mm (the actual infected RtTg region corresponds to AP ~−4.72 mm in the Mouse Brain Atlas, MBA). Coordinates used for CN injection were as followed: bregma −5.35 mm, lateral ± 2.45 mm, and dura −3.50 mm (the actual infected RtTg region corresponds to AP ~−5.88 mm in the MBA).

For manipulating the neuronal activity of RtTg in C57BL/6 J mice with optogenetics, rAAV-hSyn-hChR2(H134R)-EGFP-WPRE-pA (AAV2/9, 3.96 × 10^12 ^v.g./ml, BrainVTA) was unilaterally injected into RtTg at a volume of 250 nl, followed by optical fiber implanted 0.2 mm above the injection site. rAAV-hSyn-EGFP-WPRE-pA (AAV2/9, 2.05 × 10^12^ v.g./ml, BrainVTA) was used as a control.

For chemogenetic inactivation of RtTg neurons, 100 nl rAAV-hSyn-hM4Di-mCherry (AAV2/9, 6.42 × 10^12^ v.g./ml, Taitool) was bilaterally injected into RtTg of C57BL/6 J mice at a volume of 250 nl for each side.

For ablating RtTg neurons, a 1:1 mixture of rAAV-hSyn-Cre-EGFP-WPRE-pA (AAV2/9, 3.21 × 10^12 ^v.g./ml, BrainVTA) and rAAV-Efla-flex-taCasp3-TEVp-WPRE-pA (AAV2/9, 5.80 × 10^12 ^v.g./ml, BrainVTA) or rAAV-EF1a-DIO-EGFP-WPRE-pA (AAV2/9, 5.26 × 10^12^ v.g./ml, BrainVTA) was bilaterally injected into RtTg of C57BL/6 J mice at a volume of 250 nl for each side.

To monitor the neuronal activity of RtTg glutamatergic neurons during startle, *Vglut2-Cre* mice were unilaterally injected with 250 nl rAAV-Efla-DIO-Gcamp6m-WPRE-pA (AAV2/9, 4.75 × 10^12^ v.g./ml, BrainVTA) into the RtTg. rAAV-Efla-DIO-EGFP-WPRE-pA was used as a control.

For chemogenetic inhibition of RtTg glutamatergic neurons, rAAV-Ef1α-DIO-hM4Di-mCherry-WPRE-pA (AAV2/9, 1.72 × 10^13^ v.g./ml, Taitool) was bilaterally injected into the RtTg of *Vglut2-Cre* mice at a volume of 250 nl. rAAV-EF1a-DIO-mCherry-WPRE-pA (AAV2/9, 5.26 × 10^12^ v.g./ml, BrainVTA) was used as a control.

To dissert CN-RtTg projection, C57BL/6 J mice were unilaterally injected with 150 nl rAAV-hSyn-EGFP-WPRE-pA (AAV2/9, 2.05 × 10^12^ v.g./ml, BrainVTA) into the CN.

For anterograde transsynaptic tracing of CN-RtTg projection, *Ai9* reporter mice were unilaterally injected with 150 nl rAAV2/1-hSyn-Cre-WPRE-pA (AAV2/1, 1.03 × 10^13^ v.g./ml, Taitool) into the CN.

For optogenetic stimulation of CN-RtTg glutamatergic projection, rAAV-Ef1α-DIO-hChR2(H134R)-EGFP-WPRE-pA (AAV2/9, 3.96 × 10^12^ v.g./ml, BrainVTA) was unilaterally injected into CN of *Vglut2-Cre* mice at a volume of 150 nl, followed by optical fiber implanted 0.2 mm above the contralateral RtTg. rAAV-EF1a-DIO-EGFP-WPRE-pA (AAV2/9, 5.26 × 10^12^ v.g./ml, BrainVTA) was used as a control.

For chemogenetic inhibition of CN-RtTg glutamatergic projection, rAAV-Ef1α-DIO-hM4Di-mCherry-WPRE-pA was bilaterally injected into the CN of *Vglut2-Cre* mice at a volume of 150 nl for each side, followed by guide cannula bilaterally implanted above the RtTg. rAAV-EF1a-DIO-mCherry-WPRE-pA was used as a control.

For di-synaptic tracing of the CN-RtTg-MNs pathway, C57BL/6 J mice were unilaterally injected with 150 nl rAAV2/1-hSyn-Cre-WPRE-pA (AAV2/1, 1.03 × 10^13^ v.g./ml, Taitool) into the CN and 250 nl rAAV-Ef1α-DIO-mGFP-2A-Synaptophysin-mCherry-WPRE-pA (AAV2/9, 4.54 × 10^11^ v.g./ml, BrainVTA) into the contralateral RtTg.

For retrograde tracing of RtTg-projecting neurons, C57BL/6 J mice were unilaterally injected with 250 nl Retro-AAV-hSyn-EGFP-WPRE-pA (AAV2/R, 1.50 × 10^13^ v.g./ml, Taitool) into the RtTg.

For retrograde monosynaptic tracing of spinal MNs-projecting premotor neurons, 2 μl helper virus (Retro-AAV-hSyn-G-EGFP-WPRE-pA, AAV2/R, 1.94 × 10^13^ v.g./ml, BrainVTA) was injected into the hindlimb muscles of C57BL/6 J mice. 3 weeks later, 1 μl of the rabies virus RV-N2C-ΔG-mCherry (4.72 × 10^7^ integral field units/ml) was injected into the same hindlimb muscles. 7 days later, mice were transcardially perfused and spinal as well as brain tissues were collected for the following imaging.

Unless otherwise specified, mice were group-housed for 3 weeks after viruses injection before further conducting behaviour tests or/and immunostaining. For all experiments after surgery, the mice with incorrect virus injections and fiber/cannula implantation sites were excluded from analysis.

### C-fos detection

To perform c-fos staining of the startle reflex, mice were placed in a darkened startle chamber for the ASR test. After a 5 min habituation period, mice received 10 startle trials (different fixed white noise intensities or air puffs were used in specific experiments). Each of the trials was applied at random inter-trial intervals (ITIs) ranging from 58–63 s. Mice of the control group were placed in the chamber for 15 min without white noise stimulation. 1.5 h after being placed in the startle chamber, mice were sacrificed for c-fos immunostaining procedure.

### Electromyography (EMG) recording

To perform EMG recording of neck and hindlimb muscles during optogenetic activation of RtTg neurons, AAV-hSyn-ChR2-EGFP or AAV-hSyn -EGFP were unilaterally injected into the RtTg, followed by optical fiber implantation. Three weeks later, mice were mildly anesthetized with isoflurane (1–3%), with the neck and hindlimbs were shaved off. The ends of the electrode wires were bared for 1–2 mm and threaded through surgical needles which were then inserted into the neck and hindlimb muscles. EMG signals were then amplified and filtered (Biotex Kyoto, Japan), digitized (Power 1401), and recorded (Spike2 Software) at a sampling rate of 1000-Hz. The Spike2 data were further analyzed using custom-made MATLAB routines.

### In vivo electrophysiological recordings

Mice were head-fixed in a stereotaxic frame in which the hollow ear bars were coupled to a white noise delivery system. After craniotomy and brain surface exposure, the linear silicon electrode (NeuroNexus, USA) was inserted into the RtTg to sample the neuronal firing rate of multiple units elicited by acoustic stimuli. Mice received startle trials with varied noise intensities (60, 75, 80, 85, 90, and 95 dB). Each of the white noise stimuli was applied in random order at random inter-trial intervals (ITIs) ranging from 58-63 s. Signals were amplified, digitized (Blackrock, USA), and recorded (Nex Technologies, USA) at a sampling rate of 30 kHz. RtTg neuronal firing rate as well as firing latency after the onset of white noise were analyzed using NeuroExplore software (Plexon Inc.).

### In vivo fiber photometry

Mice were housed individually for two weeks after AAV injection and optical fiber (200 μm diameter, 0.33 numerical aperture (NA), AniLab) implantation. Fluorescence signals were output to an amplifier (C7319, Hamamatsu) with a 0.01 s moving average filter (MF525-39, Thorlabs). The signals were then digitized (Power 1401) and recorded (Spike2 Software) at a 300 Hz sampling rate. The laser power at the tip of optical fiber was 15 μW for RtTg. For monitoring the neuronal activity of RtTg during startle, we recorded white noise stimuli as well as airpuffs induced startle reflexes and Ca^2+^ transients or EGFP signals simultaneously. The raw data was then analyzed using MATLAB. Ca^2+^ fluorescence changes (*ΔF/F*) during startle reflex and conditioned fear were aligned to individual bouts and were calculated as (F−F_0_)/F_0_, where F_0_ was the average fluorescence signals from 1 s preceding stimuli (95 dB stimuli or the CS) to stimuli onset. (*ΔF/F*) values were presented as heatmaps or plotted with shaded area indicating s.e.m.

### In vivo optogenetic manipulation

Mice were housed individually for three weeks after AAV injection and optical fiber (200 μm diameter, 0.33 NA, AniLab) implantation. each optical fiber was attached to a 473-nm laser generator (AniLab-Opto), and light pulses were delivered using Master-8 (A.M.P.I.). For in vivo optogenetic activation of RtTg neurons in general or RtTg glutamatergic neurons, mice were placed in a startle chamber and received blue light stimulation (473-nm, 15 ms pulse, 5 mW, 10 mW, 15 mW, and 20 mW). For activation of CN-RtTg projection, *Vglut2-Cre* mice were placed in the startle chamber and received blue light stimulation (473 nm, 15 ms pulse, 15 mW). Each mouse received 10 stimuli in total with an inter-trial interval of 30 s, and opto-evoked startle amplitude was calculated as the mean amplitude of 10 stimuli trials.

### Electrophysiological recording

Mice were anaesthetized deeply with an intraperitoneal (i.p.) injection of pentobarbital sodium (0.5%, 0.01 ml/g) and coronal brain slices (300 μm) were sectioned using a vibratome (Leica VT1200S) after transcardially perfused with ice-cold modified *N*-methyl-d-glutamine (NMDG) artificial cerebrospinal fluid (aCSF) which contained NMDG 93 mM, KCl 2.5 mM, NaH_2_PO_4_ 1.2 mM, NaHCO_3_ 30 mM, N-2-hydroxyethylpiperazine-N-2-ethanesulfonic acid (HEPES) 20 mM, glucose 25 mM, Na-ascorbate 5 mM, Na-pyruvate 3 mM, thiourea 2 mM, MgCl_2_ 10 mM and CaCl_2_ 0.5 mM (continuously bubbled with 5% CO_2_ and 95% O_2_, pH 7.4, 300–310 mOsm). Brain slices were incubated initially in NMDG aCSF (10 min, 32 °C) before transferred to HEPES aCSF that contained NaCl 92 mM, KCl 2.5 mM, NaH_2_PO_4_ 1.2 mM, NaHCO_3_ 30 mM, HEPES 20 mM, glucose 25 mM, Na-ascorbate 5 mM, Na-pyruvate 3 mM, thiourea 2 mM, MgCl_2_ 2 mM, CaCl_2_ 2 mM (continuously bubbleed with 5% CO_2_ and 95% O_2_, pH 7.4, 300–310 mOsm) for approximately 1 h at 21–23 °C. After recovery, slices were transferred into a recording chamber and continuously perfused with HEPES aCSF (continuously bubbled with 5% CO_2_ and 95% O_2_). A fluorescent microscope (BX51WI, Olympus) with IR-DIC and a CCD camera was used to visualize target neurons.

For recording opto-evoked action potentials in ChR2^+^ neurons, whole-cell current clamp mode was obtained with pipettes (5-7 MΩ) filled with internal solution which contained K-gluconate 145 mM, MgCl_2_ 4 mM, HEPES 10 mM, EGTA 10 mM, Mg-ATP 5 mM, and Na-GTP 0.3 mM (pH 7.2, 285–290 mOsm). 473 nm blue light (5 mW/mm^2^, 5 or 10 Hz) was delivered to activate ChR2^+^ neurons.

Opto-evoked excitatory postsynaptic currents (EPSCs) were recorded with internal solution which contained K-gluconate 145 mM, MgCl_2_ 4 mM, HEPES 5 mM, EGTA 10 mM, Mg-ATP 5 mM, and Na-GTP 0.3 mM (pH 7.2, 285-290 mOsm) (pH 7.2, 285–290 mOsm). Opto-evoked inhibitory postsynaptic currents (IPSCs) were recorded with internal solution which contained CsCl 120 mM, MgCl_2_ 4 mM, HEPES 10 mM, EGTA 10 mM, Mg-ATP 5 mM, and Na-GTP 0.3 mM. Neurons were holding at −70 and 0 mV using a whole-cell voltage-clamp for recording opto-evoked EPSC and IPSC respectively. Single optical stimulation (5 ms, 10 mW/mm^2^) were delivered to ChR2^+^ terminals. 2 mM KYNA was added into the HEPES aCSF to block the opto-evoked EPSC. 2 mM KYNA and 20 μM bicuculline were added to the HEPES aCSF to block the opto-evoked IPSCs.

To determine the excitability of target neurons, whole-cell current clamp mode (*I* = *0 pA*) was obtained with pipettes (5-7 MΩ) filled with internal solution which contained K-gluconate 145 mM, MgCl_2_ 4 mM, HEPES 10 mM, EGTA 10 mM, Mg-ATP 5 mM, and Na-GTP 0.3 mM (pH 7.2, 285-290 mOsm). Depolarizing current step (1 s, −20 to 140 pA) was injected into the target neurons. CNO was added to the HEPES aCSF (continuously bubbled with 5% CO_2_ and 95% O_2_) for at least 10 min to act on hM3Dq^+^/hM4Di^+^ cell bodies or terminals. For membrane potential changes recording, CNO was bath applied for 10 min after 5 min stable baseline.

The electrophysiological signals were recorded via a MultiClamp 700B amplifier (Molecular Devices) equipped with Digidata 1550 A (Axon Instruments), digitized at 10 kHz, and filtered at 3 kHz. Data with a series resistance >25 MΩ or changed by >20% were discarded before analyzed using Clampfit 10.3 (Molecular devices, USA) and Minianalysis (Synaptosoft).

### Immunofluorescence

Mice were anaesthetized deeply with an i.p. injection of pentobarbital sodium (0.5%, 0.01 ml/g) and transcardially perfused with ice-cold PBS (0.1 M, 20 ml) followed by 4% paraformaldehyde (PFA) dissolved in 0.1 M PBS. Brains or spinal cord were removed and then post-fixed in 4% PFA (4 °C, overnight) before being transferred to 30% sucrose-PBS (4 °C, 2 days). Brains were then frozen sectioned (Leica CM 1860) into 40 μm coronal slices and collected into PBS for immunostaining. Floating sections were then washed twice in PBS, incubated in 0.5% Triton X-100 for 20 min, blocked with 10% goat or donkey serum for 40 min (room temperature), followed by primary antibodies incubation at 4 °C for at least 20 h. Primary antibodies include anti-c-fos (1:1000, Abcam, ab190289), anti-NeuN (1:500, Millipore, MAB377, clone A60), anti-glutamate (1:500, Sigma, G6642), anti-ChAT (1:200, Millipore, MAB144P), anti-GFP (1:100, Thermo Fisher, A-11122). After being washed twice in PBS, sections were incubated with secondary antibodies for 1.5 h (room temperature). Secondary antibodies included goat anti-rabbit Alexa Fluor 488 (1:1000, Cell Signaling Technology, 4412 S), goat anti-rabbit Alexa Fluor 594 (1:1000, Cell Signaling Technology, 8889 S), goat anti-mouse Alexa Fluor 594 (1:1000, Thermo Fisher, A-11032), and Donkey anti-goat Alexa Fluor 488 (1:200, Thermo Fisher, A-11055). Finally, sections were stained with DAPI (1:1000, Sigma) before being mounted under coverslips with an anti-quenching mounting medium and imaged using Zeiss LSM 880 microscope. For c-fos staining, mice were perfused 1.5 h after behavioural experiments. Cell numbers were counted automatically using ImageJ (NIH).

#### Drug infusion

For chemogenetic inhibition of RtTg neurons, C57BL/6 J/*Vglut2-Cre*/*Vgat-Cre* mice were i.p. injected with CNO (5 mg/kg, Sigma) 30 min before conducting following behaviour test.

For chemogenetic inhibition of CN-RtTg projections, mice were housed individually for 1–4 weeks after AAV injection or/and cannula implantation. Internal injectors attached to 10 μL syringes were inserted into guide cannula (RWD) for local CNO (0.2 μg/μl, 500 nl/side, Sigma) infusion at a rate of 200 nl/min using an infusion pump (KDS Scientific). After the infusion, injectors were hold still for 1 min to allow for full drug diffusion into the target region. Behaviour tests were conducted 15 min after CNO infusion.

### Statistics and reproducibility

Statistical analysis was performed using MATLAB (MathWorks) and GraphPad Prism 8.0 (GraphPad Software). Comparisons between control and treatment groups were conducted using paired/unpaired *t-tests* or two-way ANOVA combined with FDR corrections, as indicated in the figure legends. Data are presented as mean ± s.e.m, with significant difference defined as *P* < *0.05*. No statistical methods were used to predetermine sample sizes, but our sample sizes are similar to those reported in previous publications. Unless otherwise noted, all representative experiments were repeated at least three times independently with similar results.

### Reporting summary

Further information on research design is available in the Nature Research Reporting Summary linked to this article.

## Supplementary information

Supplementary Information

Description of Additional Supplementary Files

Supplementary Movie 1

Supplementary Movie 2

Reporting summary

## Data Availability

The in situ hybridization database from Allen Mouse Brain Atlas (https://mouse.brain-map.org/experiment/show/73818754) (https://mouse.brain-map.org/experiment/show/72081554) were used. The data that support the findings of this study are provided in the article and its Supplementary information files, and are available from the corresponding author upon reasonable request. Source data are provided with this paper.

## Source data

Source Data

## Code availability

The custom codes that support the findings of this study are available from the corresponding author upon reasonable request.
