# Supplementary material for: A Brainstem reticulotegmental neural ensemble drives acoustic startle reflexes
Source: Nat Commun. 2021 Nov 4;12:6403. doi: 10.1038/s41467-021-26723-9 (PMC8568936; doi:10.1038/s41467-021-26723-9)
Supplement: Supplementary file 1 — Supplementary Information [file 41467_2021_26723_MOESM1_ESM.pdf]

Supplementary information

A Brainstem Reticulotegmental Neural Ensemble Drives Acoustic Startle Reflexes

Contains: Supplementary Figures 1-24

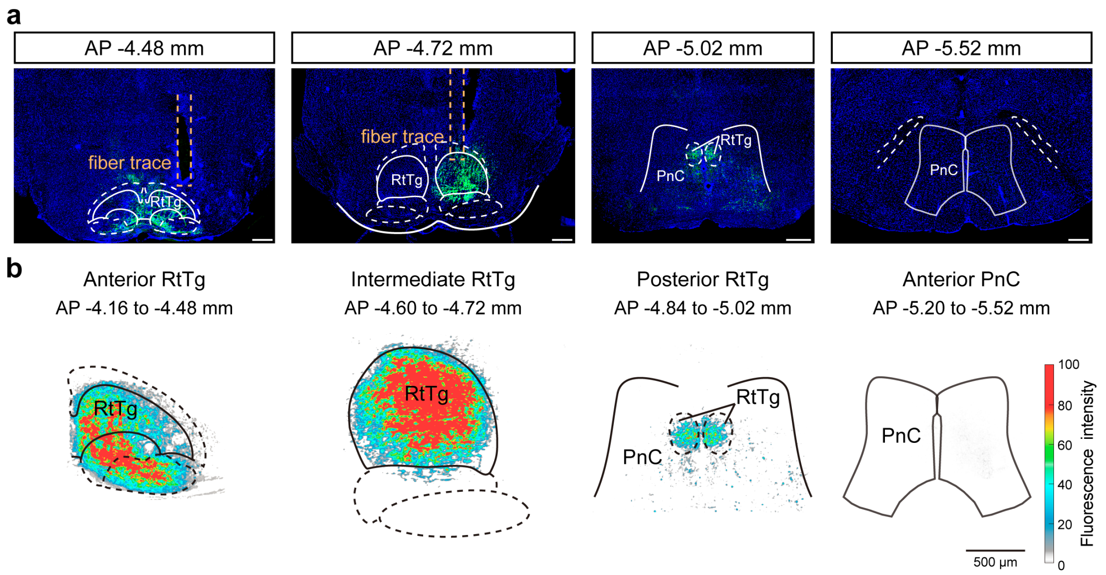

Supplementary Fig. 1. ChR2 virus injected into the RtTg did not infect the adjacent PnC region. a Representative image confirming ChR2-EGFP expression in different RtTg coronal sections as well as the absence of ChR2-EGFP expression in the PnC. Scale bar, 400 μm. **b** Heatmaps of fluorescence intensity of ChR2-EGFP in the entire RtTg and PnC regions of mice receiving AAV-hSyn-ChR2-EGFP injection into the RtTg (n = 15 mice, 405 slices in total).

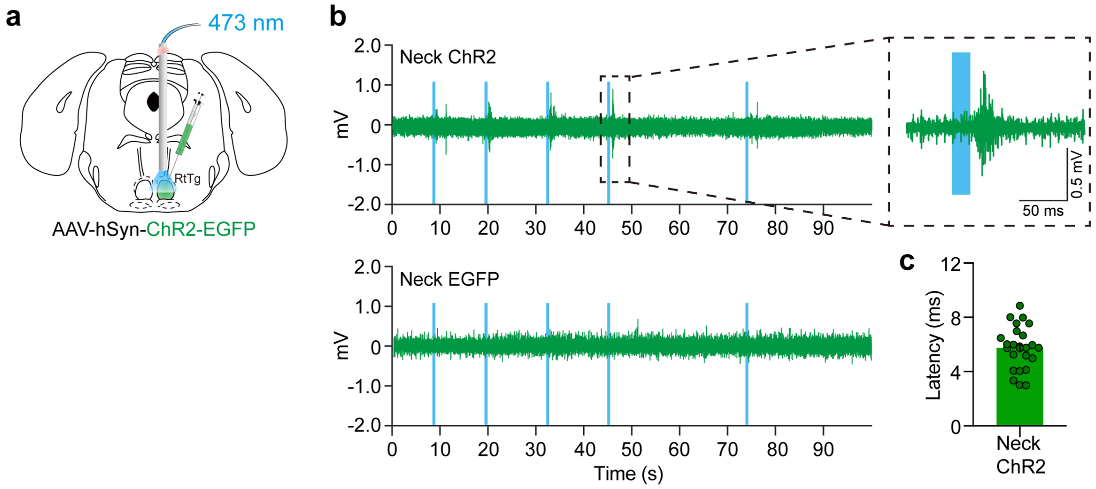

Supplementary Fig. 2. EMG recording of neck muscles during optogenetic activation of RtTg neurons. a Schematic for optogenetic activation of RtTg neurons. **b** Example EMG traces showing activities of mouse neck muscles during optogenetic activations (blue bar) of RtTg neurons. **c** Quantification of latency for neck muscle EMG activities after optogenetic activation of the RtTg (n = 5 mice, 25 bouts in total).

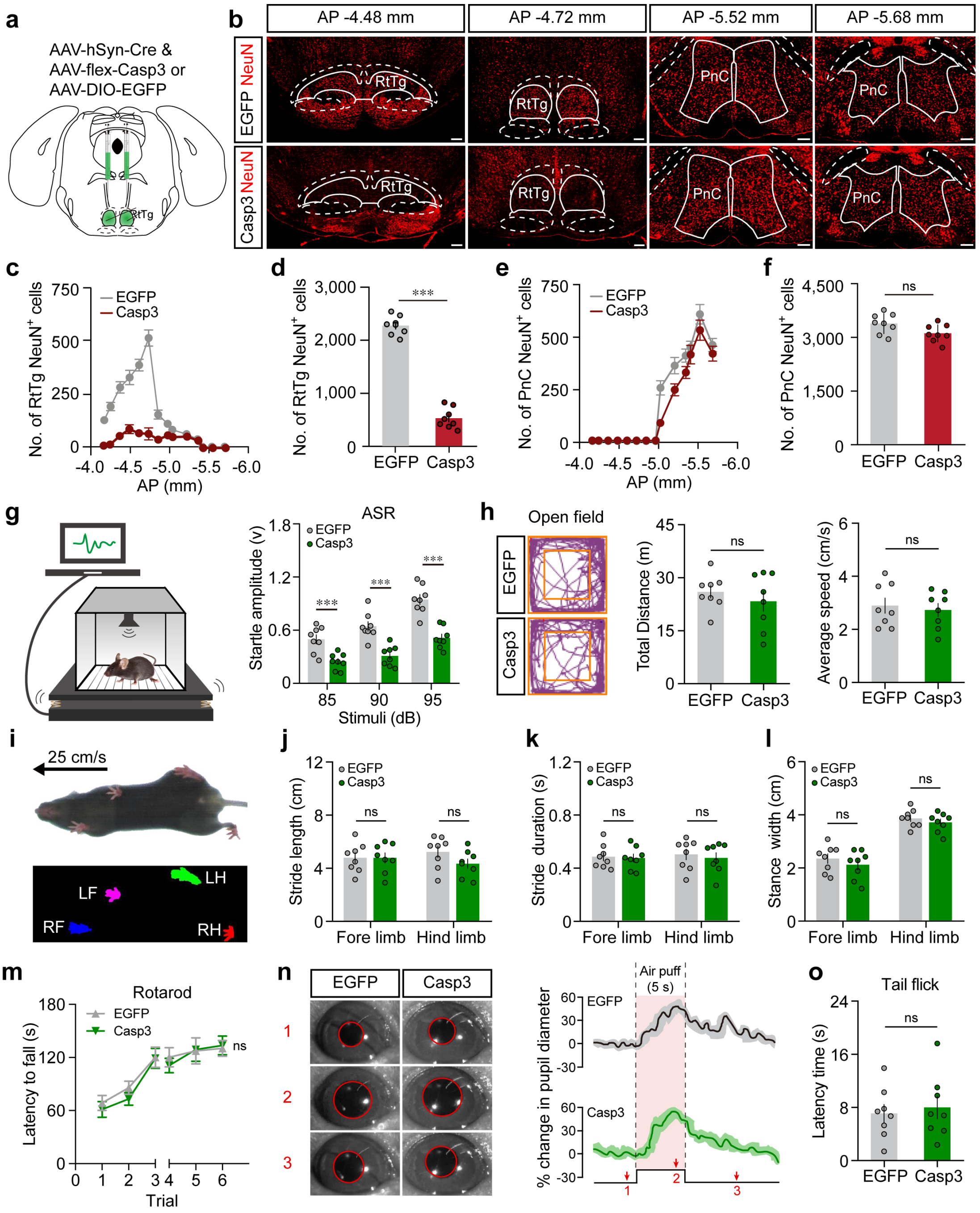

Supplementary Fig. 3. Identification of the behavioural consequences of RtTg neuronal ablation. a Schematic for Casp3-based RtTg ablation. **b** Representative images showing NeuN immunofluorescent staining in different RtTg and PnC coronal sections of a control group mouse and a lesioned group mouse. Scale bar, 300 μm. **c** Quantification of $NeuN^+$ cell numbers in consecutive RtTg coronal sections of EGFP control group mice and Casp3 lesion group mice (n = 8 per group). **d** Quantification of total number of the $NeuN^+$ cells in RtTg of EGFP control group mice and Casp3 lesion group mice

(n = 8 per group; $P = 2.47 \times 10^{-11}$). **e** Quantification of $NeuN^{+}$ cell numbers in consecutive PnC coronal sections of EGFP control group mice and Casp3 lesion group mice (n = 8 per group). **f** Quantification of total number of the $NeuN^{+}$ cells in PnC of EGFP control group mice and Casp3 lesion group mice (n = 8 per group; $P = 0.0562$). **g** Left: schematic for the ASR paradigm. Right: Quantitative graph showing the effect of RtTg lesion on ASR amplitudes (n = 8 per group; $t = 3.802$, $P = 0.0005$ (85 dB); $t = 5.065$, $P = 8.63 \times 10^{-6}$ (90 dB); $t = 6.570$, $P = 6.02 \times 10^{-8}$ (95 dB)). **h** Left: representative movement track of an EGFP control mouse and a Casp3 lesion mouse. Quantification of total distances (middle, n = 8 per group; $P = 0.4386$) and average speed (right, n = 8 per group; $P = 0.6735$) during open field test. **i** Representative images (top) showing a mouse walking on treadmill and paw prints (bottom) detected from a video settled under the treadmill. **j-l** Digi Gait analysis for EGFP control group mice and Casp3-based RtTg lesion group mice (n = 8 per group; for **j**: $t = 0.0001$, $P = 0.9999$ (fore limb); $t = 1.602$, $P = 0.1204$ (hindlimb); for **k**: $t = 0.1952$, $P = 0.8466$ (fore limb); $t = 0.5240$, $P = 0.6044$ (hindlimb); for **l**: $t = 1.054$, $P = 0.3011$ (fore limb); $t = 0.7526$, $P = 0.4580$ (hindlimb)). **m** Latency to fall on accelerating rotarod for EGFP control group mice and Casp3 lesion group mice (n = 8 per group; $F = 0.2$, $P = 0.9251$). **n** Representative video frame images showing pupil diameters (left, red circles) of an EGFP control mouse and a Casp3 lesion mouse acquired at the timepoints indicated in the pupil recording traces (right). **o** Tail-flick latency of EGFP control group mice and Casp3 lesion group mice (n = 8 per group; $P = 0.6840$). Error bar represent mean ± s.e.m. Significance was assessed using two-sided unpaired *t-tests* in **d**,**f**,**h,o,** and two-way ANOVA combining with FDR corrections in **g, j-m**. ** $P < 0.01$; *** $P < 0.001$; ns, not significant.

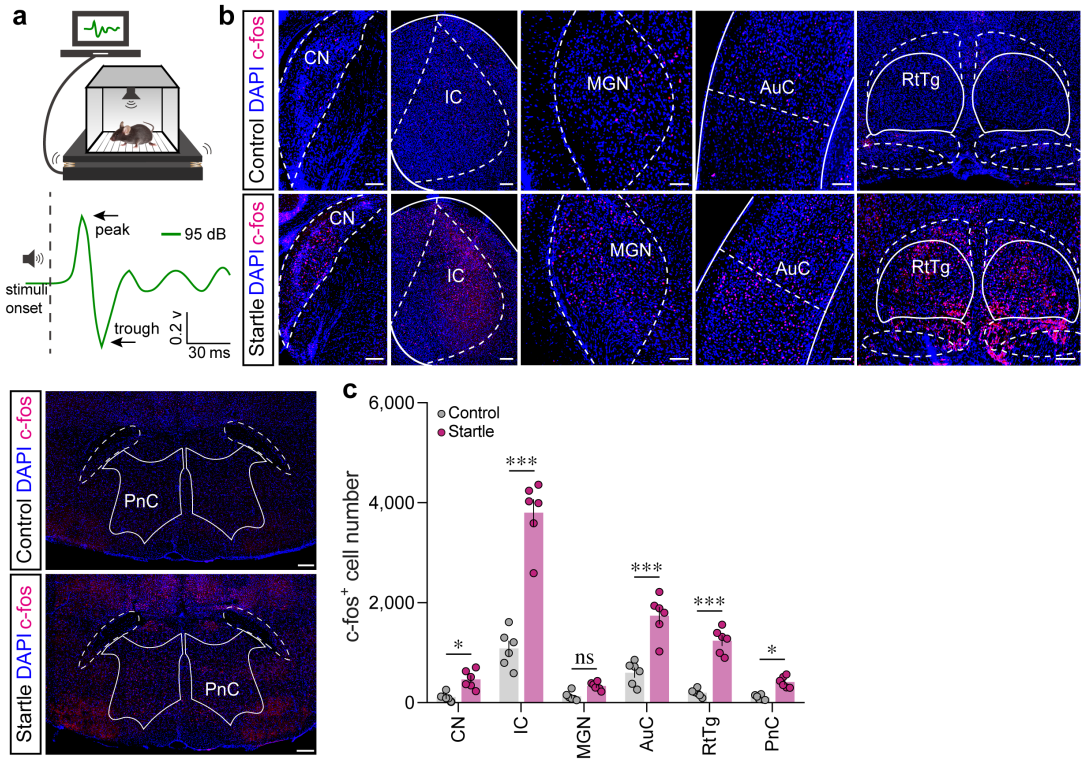

Supplementary Fig. 4. Differential c-fos expression in various brain regions of mice during ASR. a Schematic for the ASR apparatus, and the sample traces of startle responses elicited by 95 dB acoustic stimulus. The ASR amplitude was defined as the largest peak-to-trough response occurred within 200 ms after the onset of the startle stimulus. **b, c** Representative images (**b**) and quantitative analysis (**c**) of c-fos immunofluorescence in various brain regions of startled mice vs control mice (n = 6 per group; for CN, t = 2.204, P = 0.0314; for IC, t = 17.07, $P = 1.02 \times 10^{-16}$; for MGN, t = 1.298, P = 0.1991; for AuC, t = 7.171, $P = 1.28 \times 10^{-9}$; for RtTg, t = 6.566, $P = 1.37 \times 10^{-8}$; for PnC, t = 2.559, P = 0.0130). Scale bar, 200 μm. Error bar represent mean ± s.e.m. * $P < 0.05$; ** $P < 0.01$; *** $P < 0.001$; ns, not significant based on two-way ANOVA combining with FDR corrections. CN, cochlear nucleus; IC, inferior colliculus; MGN, medial geniculate nucleus; AuC, auditory cortex; RtTg, reticulotegmental nucleus; PnC, caudal pontine reticular nucleus.

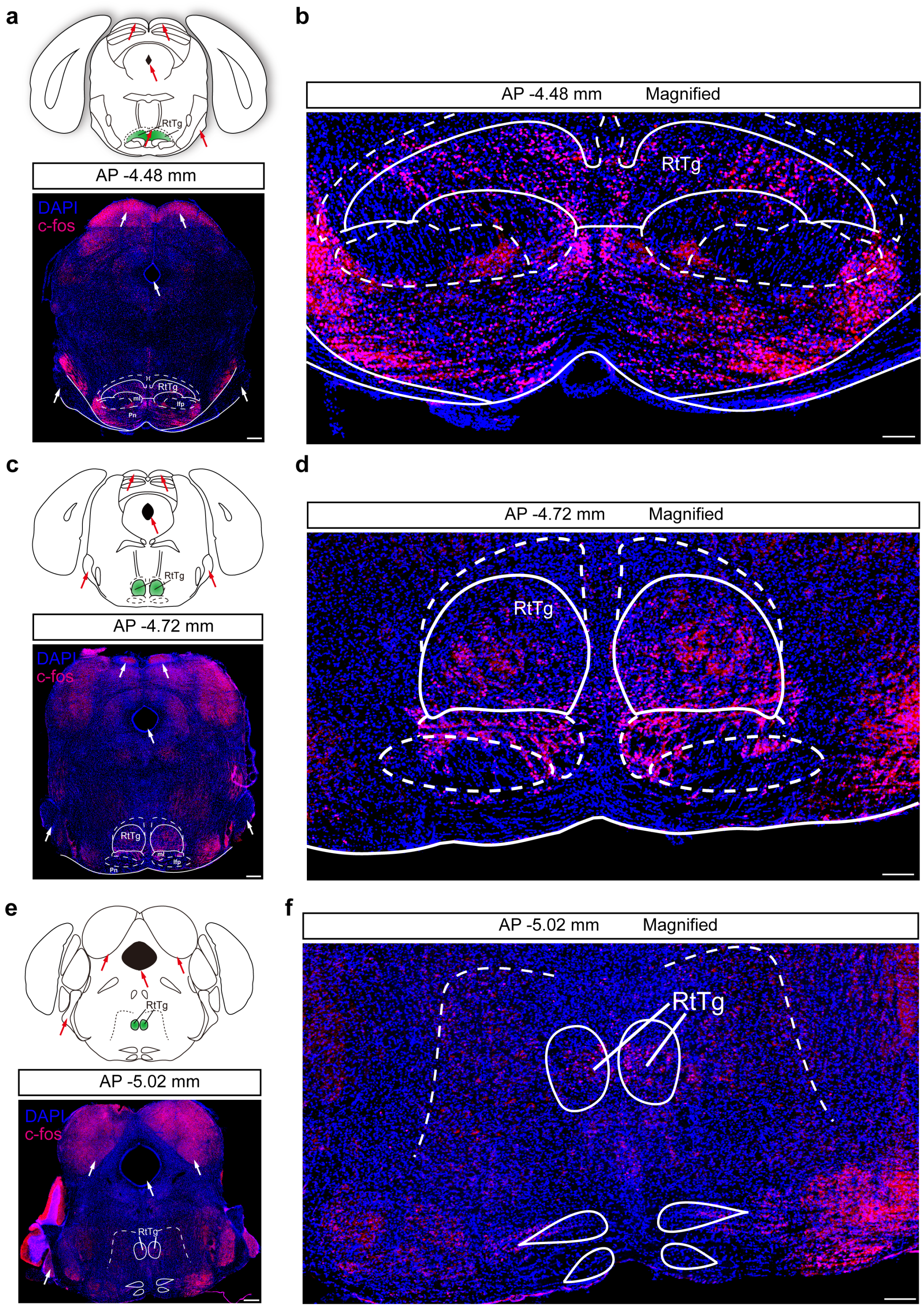

Supplementary Fig. 5. C-fos expression in different coronal sections of the RtTg during ASR. a Top: schematic of RtTg coronal section at AP -4.48 mm. Bottom: Representative image showing 95 dB acoustic stimuli-induced c-fos expression in the RtTg at AP -4.48 mm. Scale bar, 400 μm. **b** Magnified RtTg region in Supplementary Fig. 5**a**. Scale bar, 200 μm. **c** Top: schematic of RtTg coronal section at AP -4.72 mm. Bottom: Representative image showing

95 dB acoustic stimuli-induced c-fos expression in the RtTg at AP -4.72 mm. Scale bar, 400 μm. **d** Magnified RtTg region in Supplementary Fig. 5**c**. Scale bar, 200 μm. **e** Top: schematic of RtTg coronal section at AP -5.02 mm. Bottom: Representative image showing 95 dB acoustic stimuli-induced c-fos expression in the RtTg at AP -5.02 mm. Scale bar, 400 μm. Arrows indicates referable landmarks. **f** Magnified RtTg region in Supplementary Fig. 5**e**. Scale bar, 300 μm.

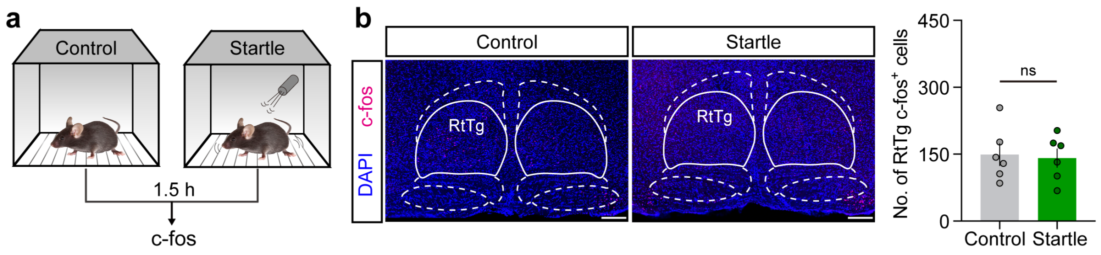

Supplementary Fig. 6. C-fos expression in the RtTg of mice during air puff-induced startle reflexes. a Schematic depiction for examining RtTg c-fos expression under air puff-induced startle reflexes. **b** Representative images (left) and quantitative analysis (right) of c-fos immunofluorescence in the RtTg of startled mice vs control mice (n = 6 per group; *P* = 0.8163). Scale bar, 200 μm. Error bar represent mean ± s.e.m. Significance was assessed using two-sided unpaired *t-tests*. ns, not significant.

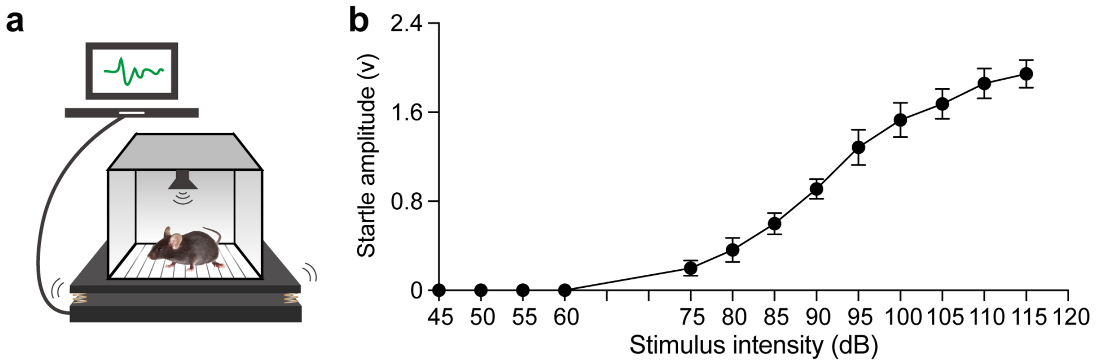

Supplementary Fig. 7. Startle responses under acoustic stimuli with different intensities. **a** Schematic for the ASR paradigm. **b** Quantitative amplitudes of ASR in response to increasing sound stimulus intensity (n = 12 mice). Error bar represent mean ± s.e.m.

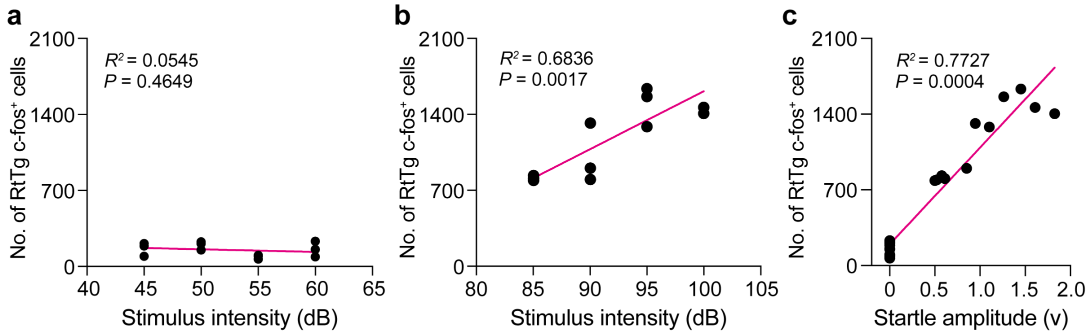

Supplementary Fig. 8. Relationship between RtTg activity and startle behaviour. a, b Correlation between low stimulus intensity (**a**, n = 12 mice)/startle-eliciting intensity (**b**, n = 11 mice) and RtTg c-fos$^+$ cell number. **c** Linear regression analysis for startle amplitude and RtTg c-fos$^+$ cell number (n = 23 mice).

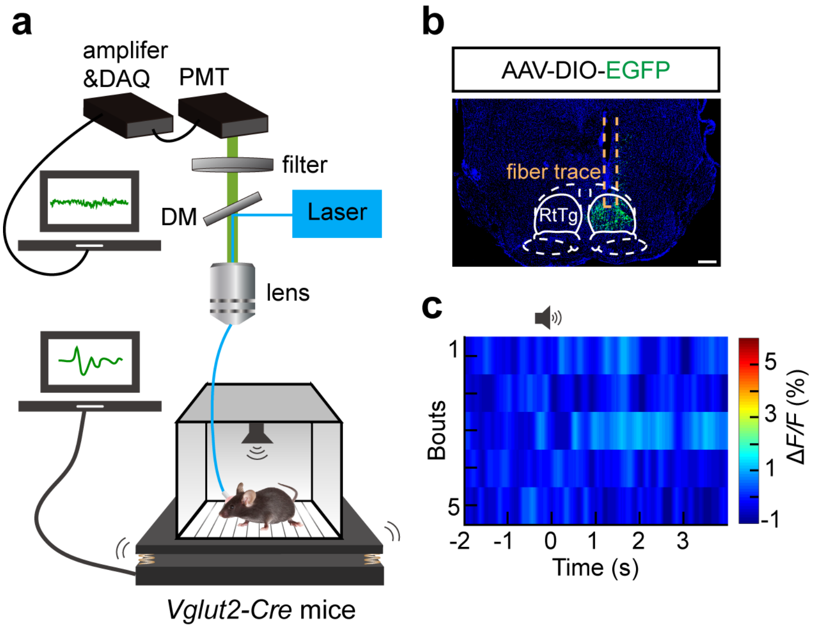

Supplementary Fig. 9. *In vivo* fiber photometry of RtTg expressing EGFP as the control. a Schematic for the *in vivo* fiber photometry apparatus. **b** A representative image confirming EGFP expression in the RtTg, and the optical fiber trace. Scale bar, 400 μm. **c** Heat map of the RtTg EGFP fluorescence signal changes aligned to the acoustic stimuli onset.

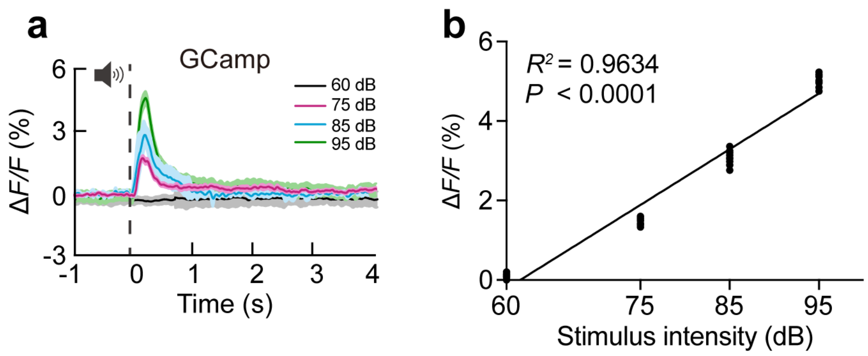

Supplementary Fig. 10. Correlation between RtTg activity and acoustic stimulus intensity. a Quantifications of calcium signal changes of RtTg glutamatergic neurons aligned to the acoustic stimuli onset (n = 9 bouts from 3 mice for each sound intensity). Thick line indicates mean and the area shaded in lighter color indicates s.e.m. **b** Linear regression analysis for RtTg glutamatergic neuronal activities and acoustic stimulus intensities (n = 9 bouts from 3 mice for each sound intensity).

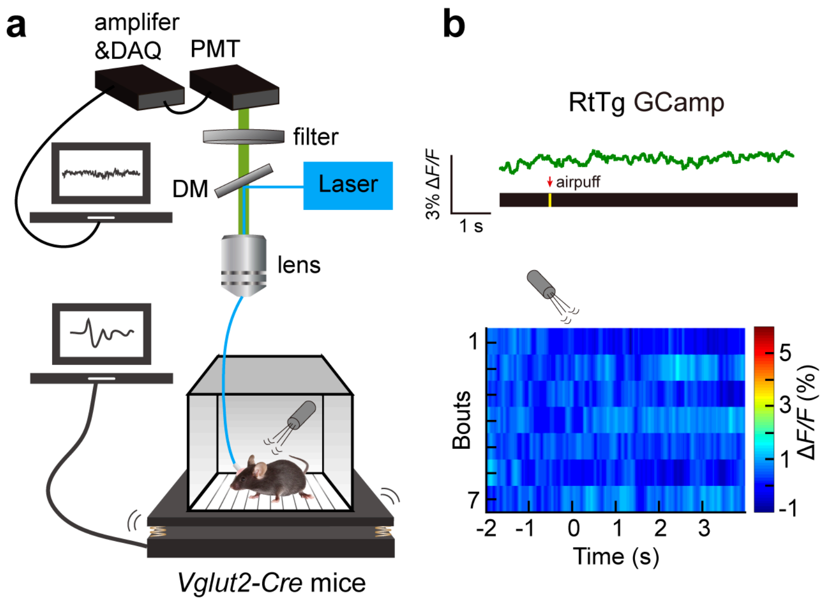

Supplementary Fig. 11. *In vivo* fiber photometry of RtTg glutamatergic neuron activity during air puff-induced startle reflexes. a Schematic for the *in vivo* fiber photometry apparatus. **b** Representative traces (top) and heat map (bottom) of calcium signal changes of RtTg glutamatergic neurons aligned to the air puff stimulus onset.

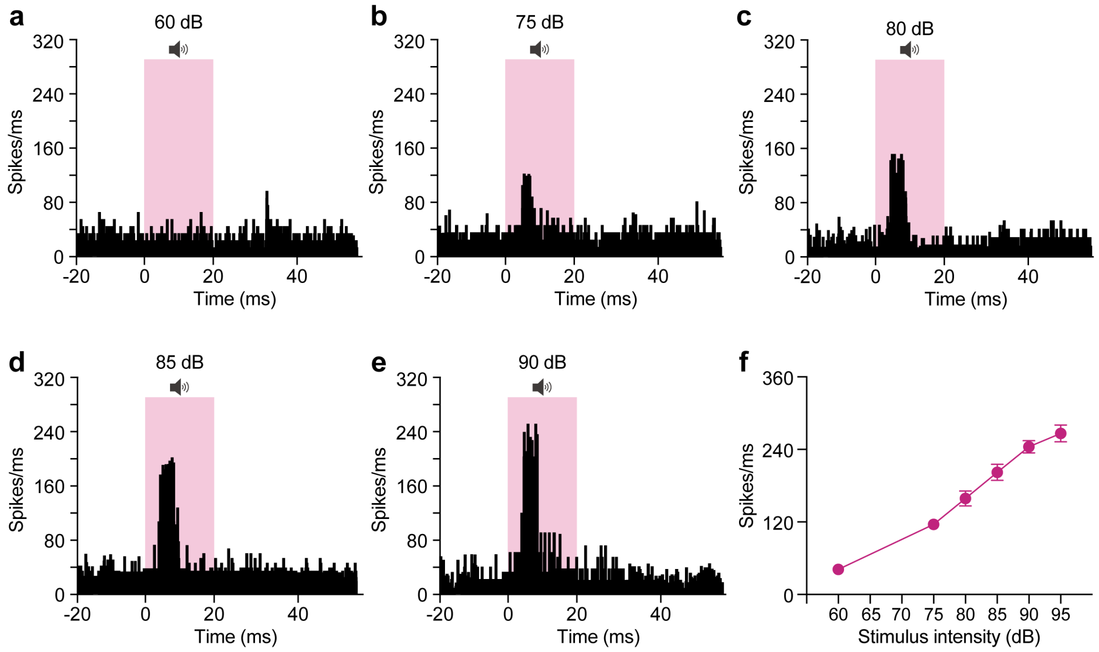

Supplementary Fig. 12. *In vivo* recordings of RtTg neuronal firings in response to acoustic stimuli. a-e The average peristimulus time histogram showing RtTg neuronal activities evoked by white noise stimuli of different intensities including 60 (**a**), 75 (**b**), 80 (**c**), 85 (**d**) and 90 dB (**e**). **f** Mean firing rate of RtTg neurons in response to different acoustic stimulus intensities (n = 5 mice). Error bar represent mean ± s.e.m.

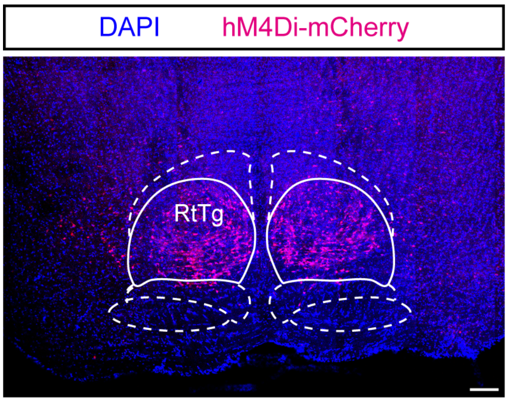

Supplementary Fig. 13. Representative image confirming hM4Di-mCherry expression in the RtTg of *Vglut2-Cre* mice. Scale bar, 300 μm.

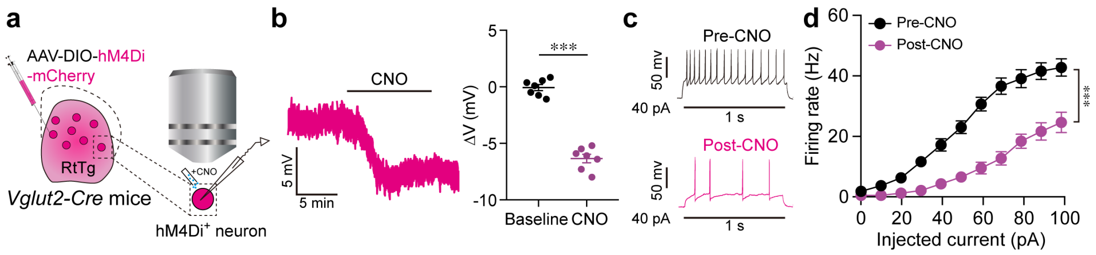

Supplementary Fig. 14. Validation of the efficiency of the chemogenetic inhibition of RtTg glutamatergic neurons. a Schematic for the chemogenetic inhibition and the whole-cell patch clamp recording of RtTg hM4Di-mCherry$^+$ neurons in the RtTg brain slice of *Vglut2-Cre* mice receiving injection of the AAV-DIO-hM4Di-mCherry into the RtTg. **b** Sample trace (left) and quantitative analysis (right) showing decreased membrane potentials in RtTg hM4Di-mCherry$^+$ neurons in the presence of CNO (n = 7 cells from 2 mice; $P = 1.11 \times 10^{-5}$). **c** Sample traces showing action potentials recorded in RtTg hM4Di-mCherry$^+$ neurons before and after bath application of CNO. **d** Quantitative analysis of the firing rate of action potentials in RtTg hM4Di-mCherry$^+$ neurons before and after bath application of CNO (n = 8 cells from 3 mice; $F = 38.09$, $P = 2.42 \times 10^{-5}$). Error bar represent mean ± s.e.m. *** $P < 0.001$ based on two-sided paired *t-tests* in **b**, and two-way ANOVA combining with FDR corrections in **d**.

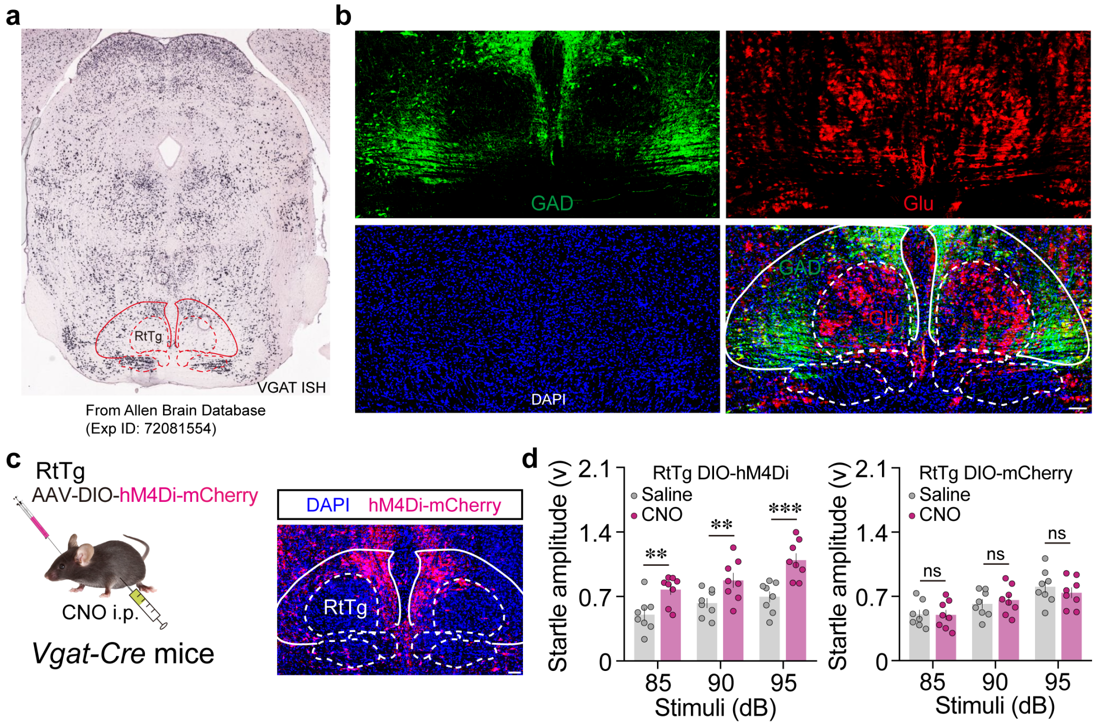

Supplementary Fig. 15. The effect of chemogenetic inactivation of RtTg GABAergic neurons on ASR. a *VGAT* mRNA in situ hybridization data in RtTg coronal section (from Allen Brain Database). **b** Representative images showing the distinct localization of GAD^+ neurons and $glutamate^+$ neurons in the RtTg. Scale bar, 100 μm. **c** Left: schematic for chemogenetic inactivation of GABAergic neurons in the RtTg of *Vgat-Cre* mice. Right: representative image confirming hM4Di-mCherry expression. Scale bar, 100 μm. **d** ASR amplitudes of *Vgat-Cre* mice receiving the injection of AAV-DIO-hM4Di-mCherry or AAV-DIO-mCherry into the RtTg, followed by i.p. CNO or saline administration (n = 8 per group; for **d** left, t = 3.177, P = 0.0028 (85 dB); t = 2.771, P = 0.0083 (90 dB); t = 4.461, $P = 5.6 \times 10^{-5}$ (95 dB); for **d** right, t = 0.0867, P = 0.9313 (85 dB); t = 0.5341, P = 0.5961 (90 dB); t = 0.8184, P = 0.4178 (95 dB)). Error bar represent mean ± s.e.m. Significance was assessed using two-way ANOVA combining with FDR corrections. ** P < 0.01; *** P < 0.001; ns, not significant.

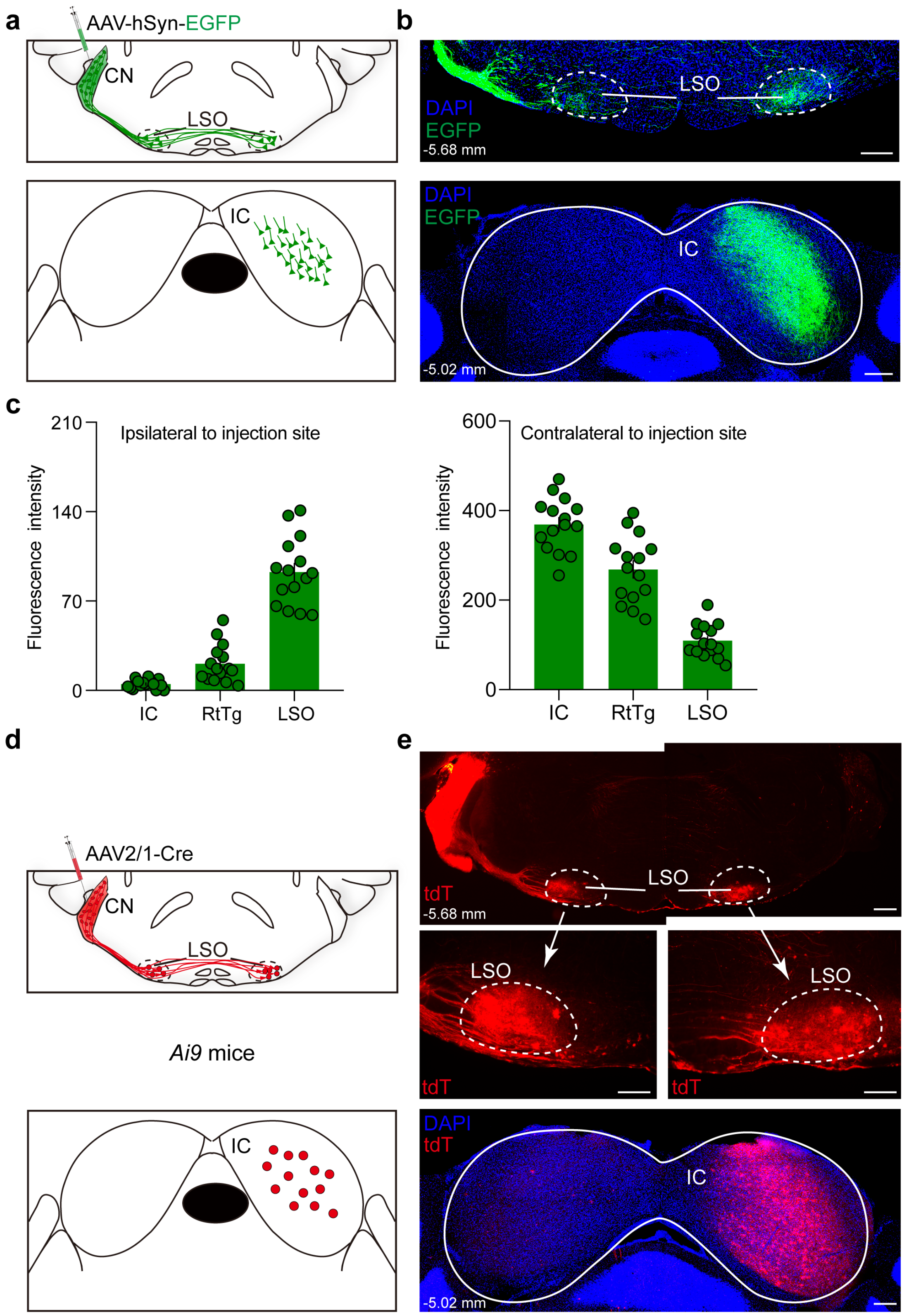

Supplementary Fig. 16. Identification of the brain regions receiving direct CN neuronal projections. a Schematic for the anterograde tracing from CN to the LSO and the IC by injecting AAV-hSyn-EGFP into the CN. **b** Representative images showing the fluorescent signal of EGFP^{+} presynaptic terminals in the LSO and the IC. Scale bar, 250 μm. **c** Quantitative fluorescence intensity of CN

EGFP$^+$ neuronal terminals in ipsilateral nuclei (IC, RtTg, LSO) and contralateral nuclei (IC, RtTg, LSO) (n = 15 mice). **d** Schematic for the anterograde transsynaptic tracing from CN to LSO and IC by injecting AAV2/1-Cre into the CN of *Ai9* (RCL-tdT) reporter mice. **e** Representative images showing the fluorescent signal of tdT$^+$ cells in the LSO and the IC. Error bar represent mean ± s.e.m. CN, cochlear nucleus; LSO, lateral superior olivary nucleus; IC, inferior colliculus. Scale bar, 250 μm.

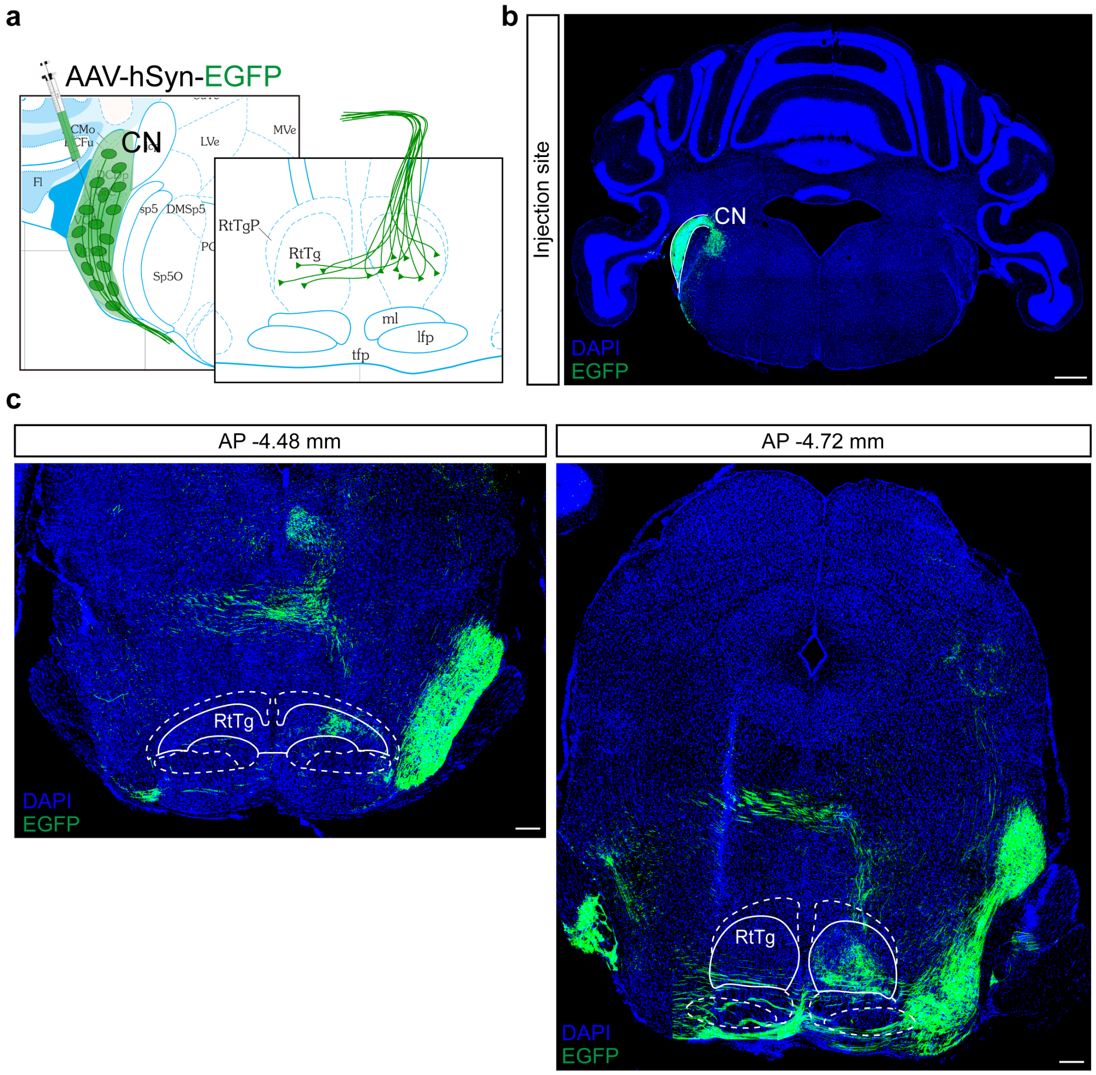

Supplementary Fig. 17. Anterograde tracing from CN to RtTg. a Schematic for the anterograde tracing from CN to RtTg by injection of an AAV-hSyn-EGFP into the CN. **b** Representative images showing CN injection site. Scale bar, 400 μm. **c** Representative images showing the fluorescent signal of $EGFP^+$ presynaptic terminals in different RtTg coronal sections. Scale bar, 300 μm.

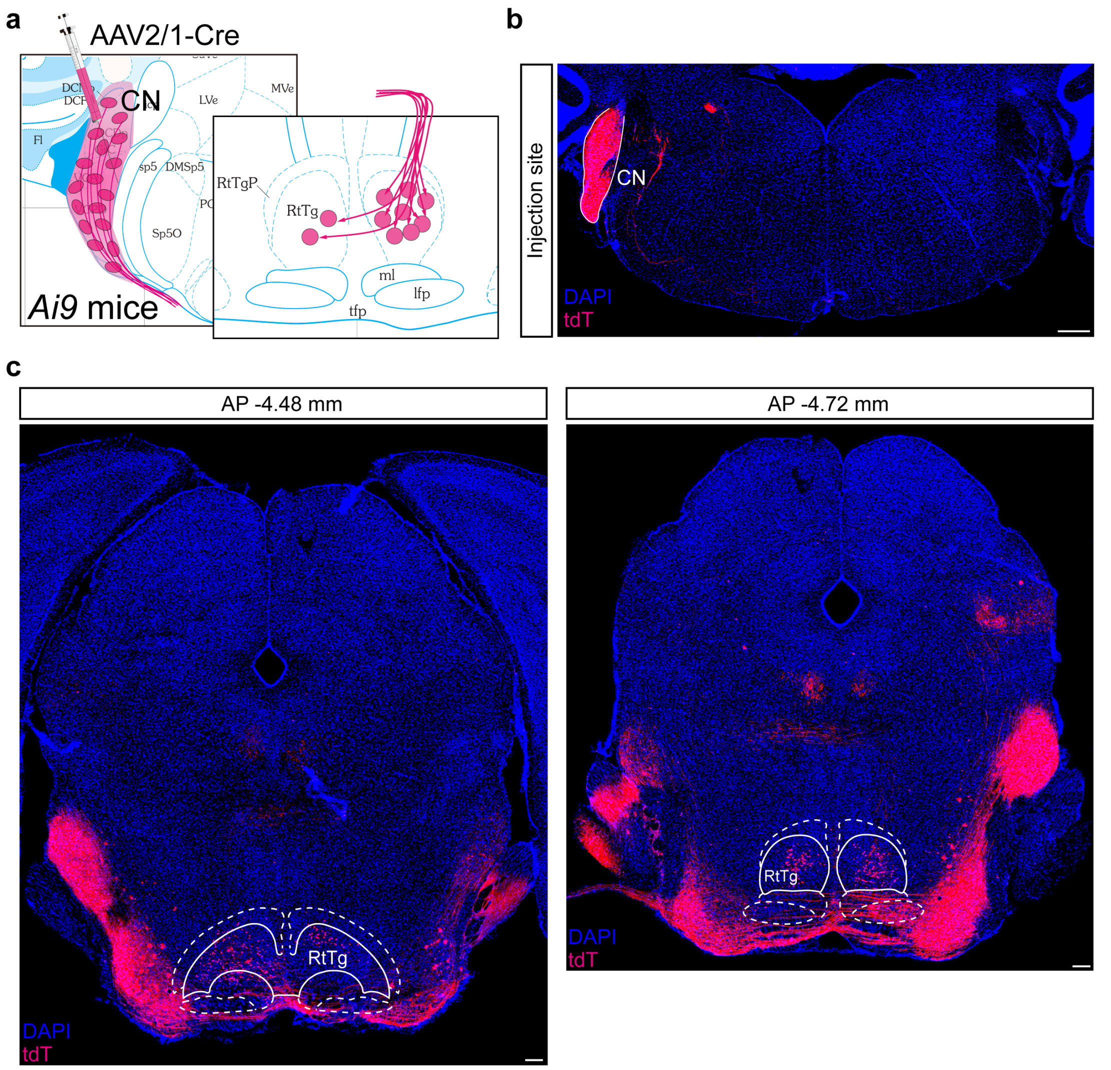

Supplementary Fig. 18. Anterograde Transsynaptic Tracing from the CN to the RtTg. a Schematic for the anterograde mono-transsynaptic tracing from CN to RtTg by injecting AAV2/1-Cre into the CN of *Ai9* (RCL-tdT) reporter mice. **b** Representative images showing CN injection site. Scale bar, 200 μm. **c** Representative images showing tdT^+ neurons in different RtTg coronal sections. Scale bar, 300 μm.

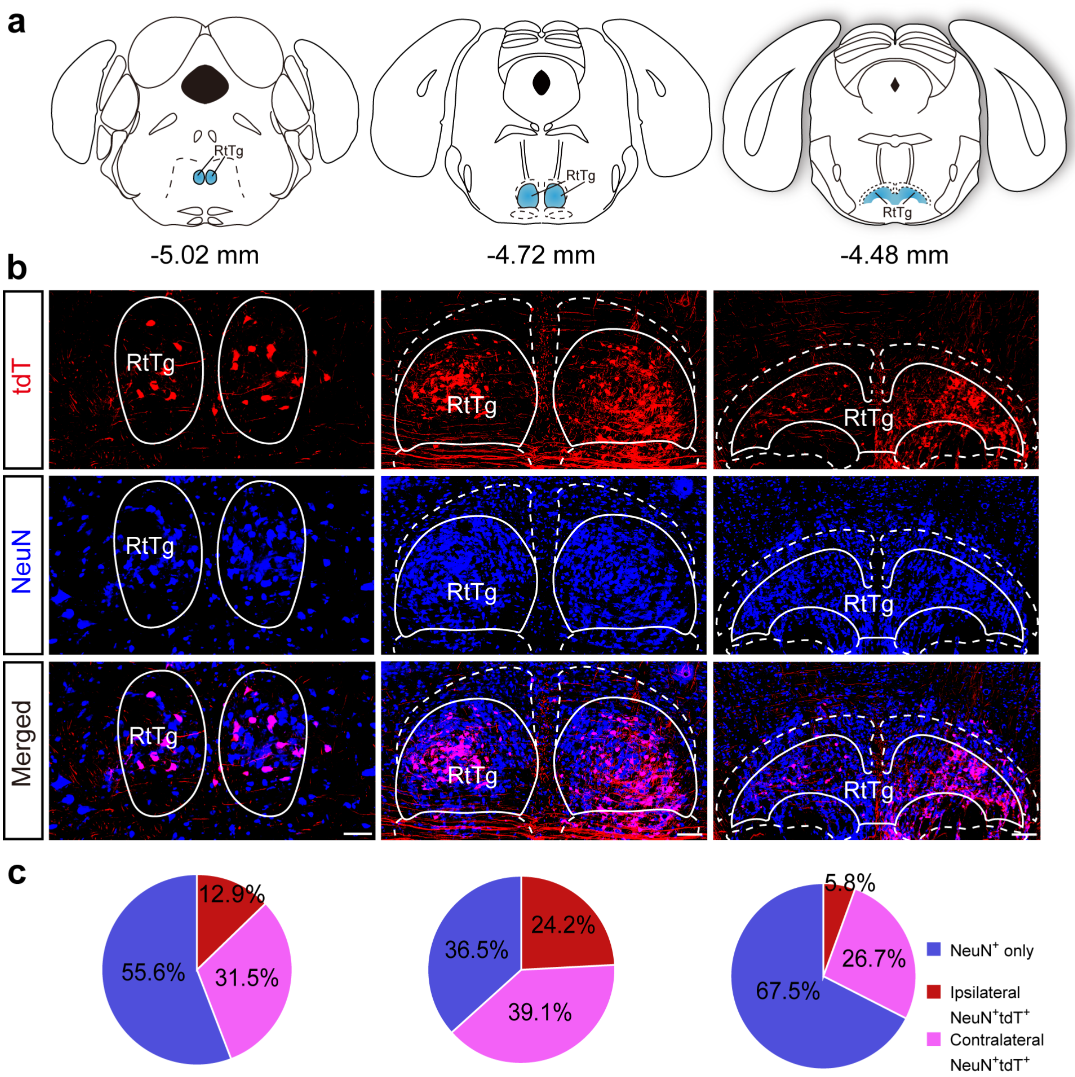

Supplementary Fig. 19. Identification of RtTg neurons receiving CN neural projections. a RtTg Atlas of different coronal sections. **b** Representative images showing tdT$^+$ RtTg neurons co-labelled with NeuN in *Ai9* (RCL-tdT) reporter mice receiving intra-CN injection of AAV2/1-Cre. Scale bar, 200 μm. **c** Pie charts indicating the percentage of ipsilateral and contralateral NeuN$^+$ RtTg neurons co-labelled with or without tdT in different RtTg coronal sections.

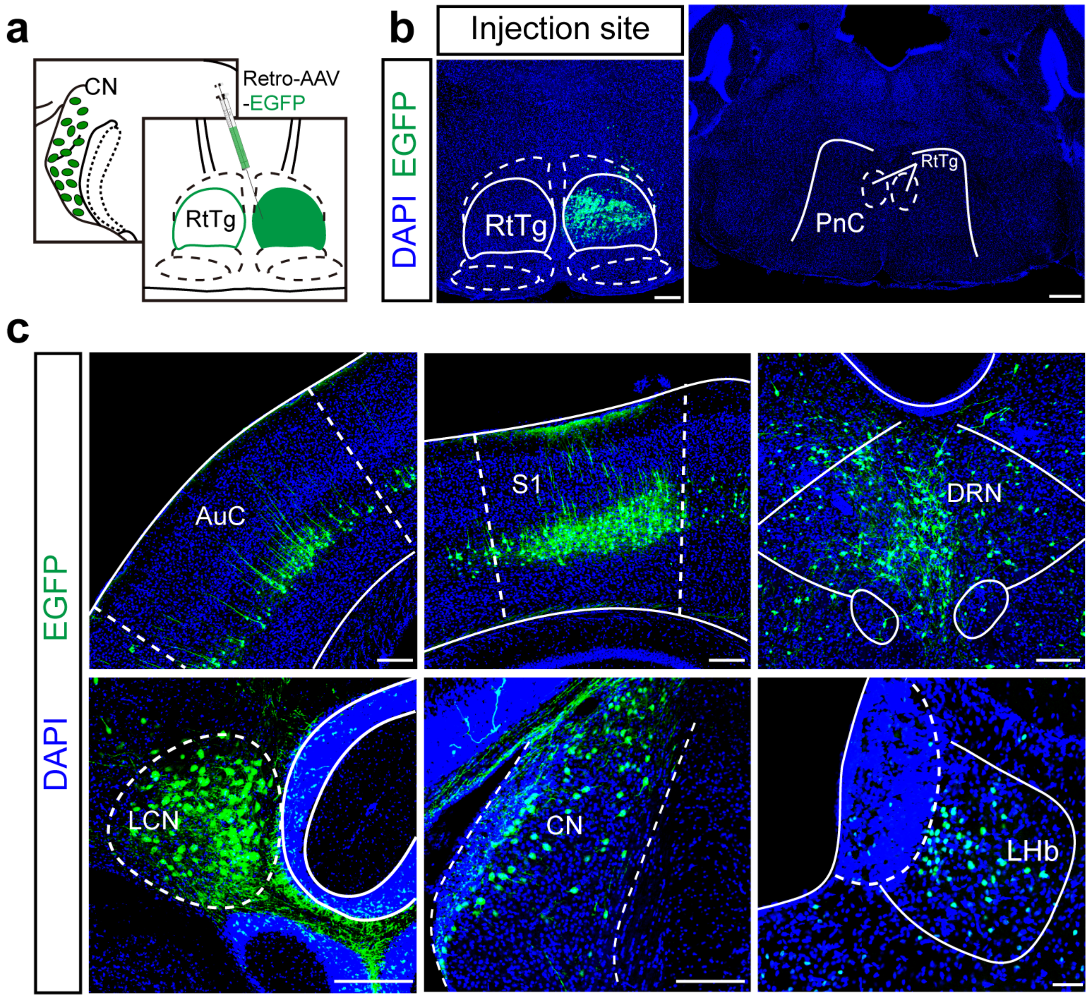

Supplementary Fig. 20. Retro-AAV-EGFP virus injected into the RtTg did not infect the adjacent PnC region. a Schematic for the retrograde tracing from RtTg to CN by injecting Retro-AAV-EGFP into the RtTg. **b** Left: representative images showing RtTg injection site. Scale bar, 200 μm. Right: representative image showing the absence of retro-AAV-EGFP expression in the PnC. Scale bar, 400 μm. **c** Representative images showing retrogradely labeled EGFP^{+} neurons in different brain nuclei (n = 8 mice). Scale bar, 200 μm. AuC, auditory cortex; S1, primary somatosensory cortex; DRN, dorsal raphe nucleus; LCN, lateral cerebellar nucleus; CN, cochlear nucleus; LHb, lateral habenular nucleus.

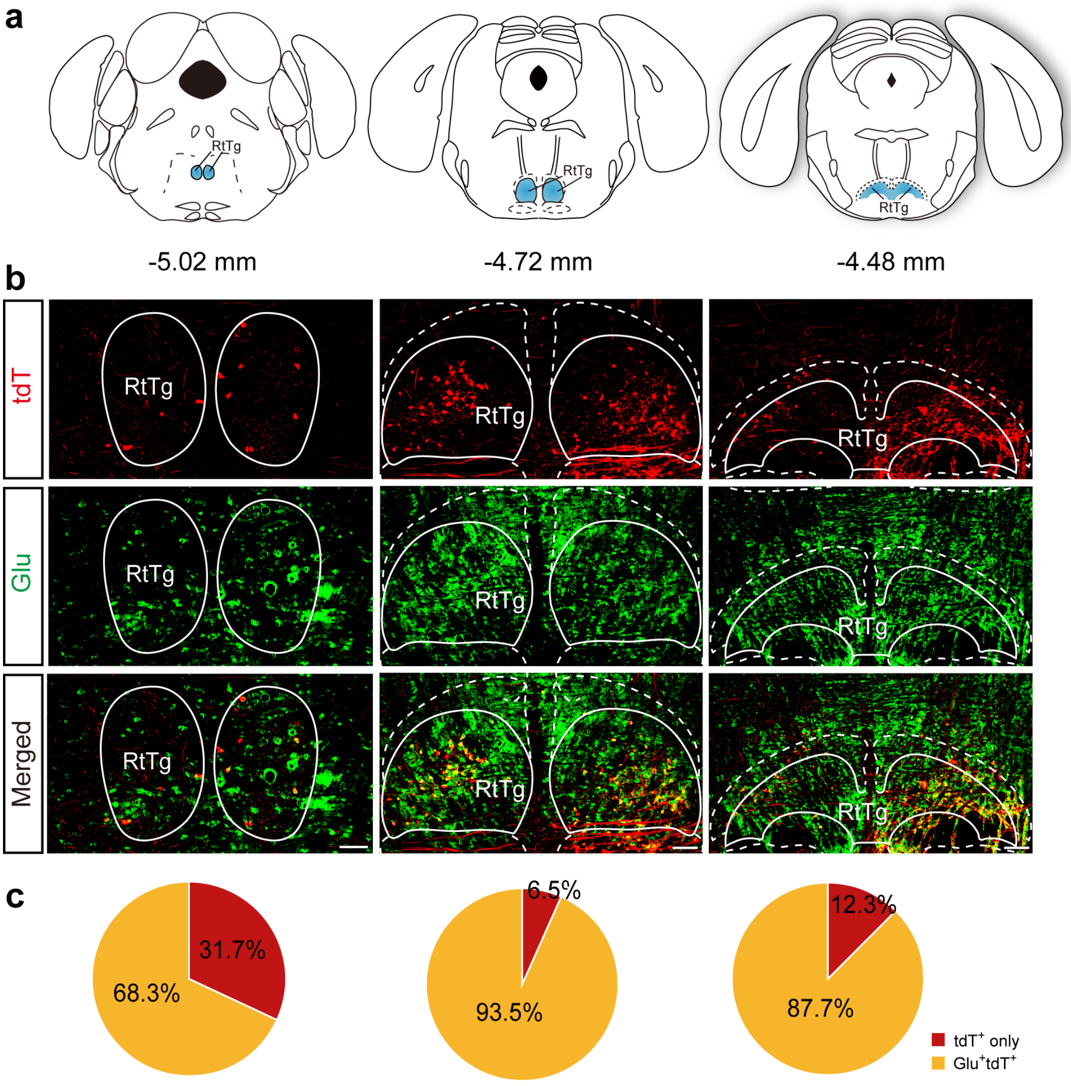

Supplementary Fig. 21. Identification of RtTg glutamatergic neurons receiving CN neural projections. a RtTg Atlas of different coronal sections. **b** Representative images showing tdT^{+} RtTg neurons co-labelled with glutamate in *Ai9* (RCL-tdT) reporter mice receiving intra-CN injection of AAV2/1-Cre. Scale bar, 200 μm. **c** Pie charts indicating the percentage of ipsilateral and contralateral tdT^{+} RtTg neurons co-labelled with or without glutamate in different RtTg coronal sections.

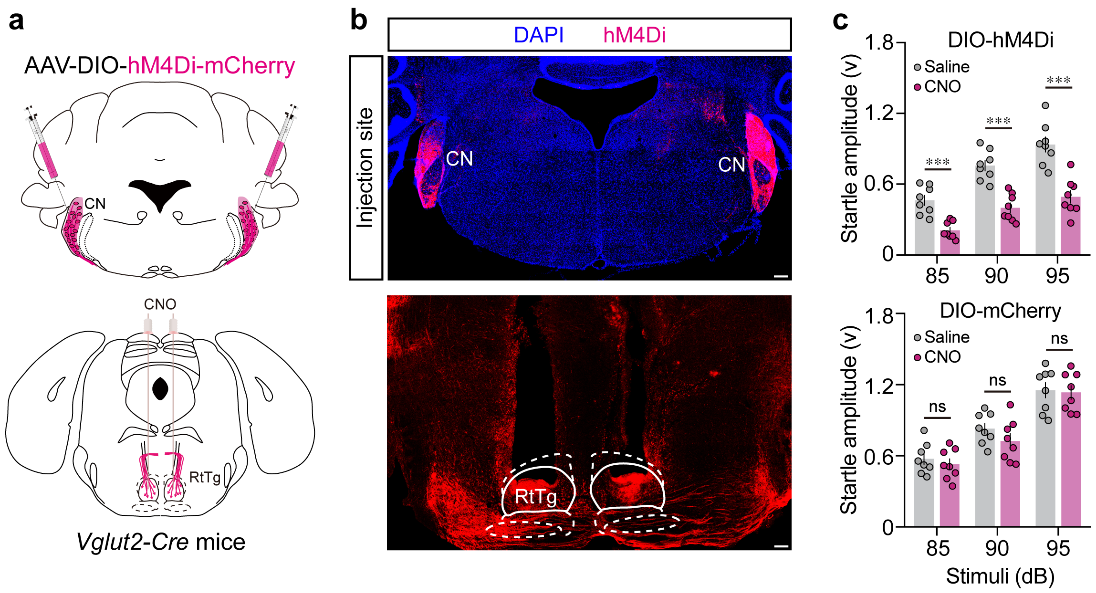

Supplementary Fig. 22. The effect of chemogenetic inhibition of CN-RtTg projections on ASR. a Schematic for the chemogenetic inhibition of CN-RtTg glutamatergic projections by injecting Cre-dependent hM4Di virus into the CN of *Vglut2-Cre* mice, followed by implantation of guide cannula above the RtTg. **b** Representative images showing CN injection sites as well as hM4Di-mCherry^{+} terminals in the RtTg. Scale bar, 200 μm. **c** ASR amplitudes of *Vglut2-Cre* mice receiving bilateral injection of AAV-DIO-hM4Di-mCherry or control AAV-DIO-mCherry into the CN, followed by intracranial delivery of CNO (0.2 μg/μl) or saline into the RtTg (n = 8 per group; for **c** top, t = 3.839, P = 0.0004 (85 dB); t = 5.391, P = 2.97 × 10^{-6} (90 dB); t = 6.706, P = 3.83 × 10^{-8} (95 dB); for **c** bottom, t = 0.5932, P = 0.5562 (85 dB); t = 1.365, P = 0.1796 (90 dB); t = 0.2356, P = 0.8149 (95 dB)). Error bar represent mean ± s.e.m. Significance was assessed using two-way ANOVA combining with FDR corrections. *** P < 0.001; ns, not significant.

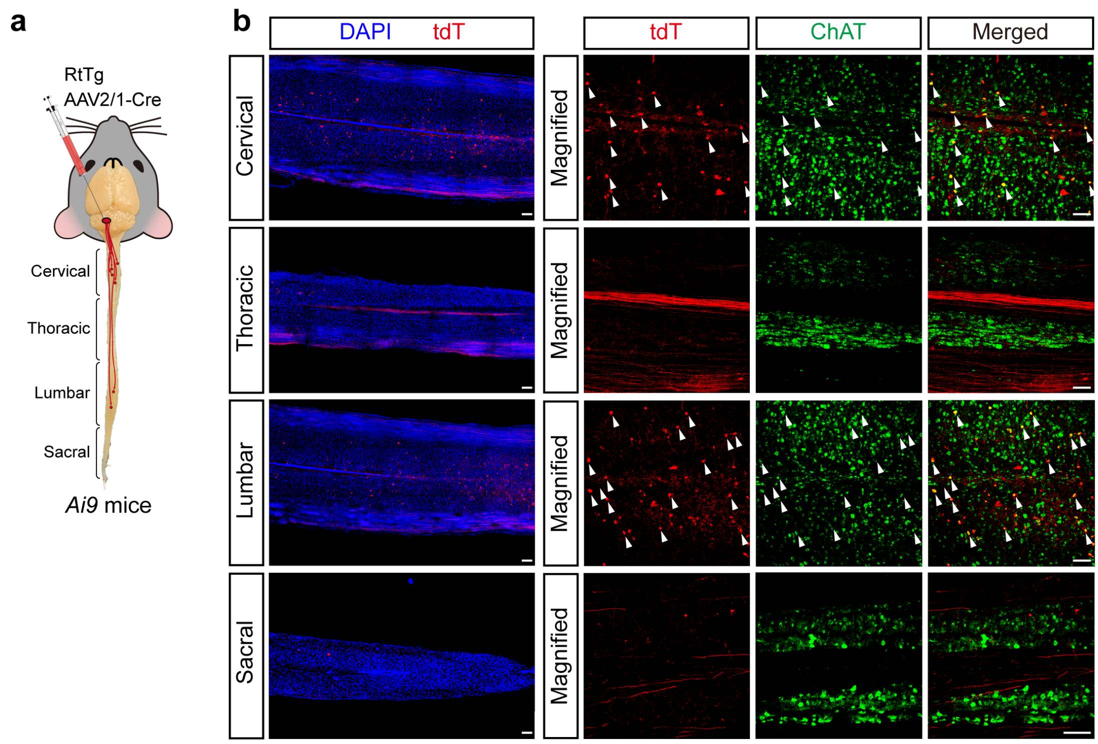

Supplementary Fig. 23. Identification of spinal cord segments receiving RtTg neuronal projections. a Schematic for the AAV2/1-Cre-based anterograde transsynaptic tracing from the RtTg to multiple spinal cord segments. **b** Representative images showing the tdT^{+} neuron labelling in transverse sections of spinal segments, and the co-labelling of tdT with ChAT (white arrowheads). Scale bar, 100 μm.

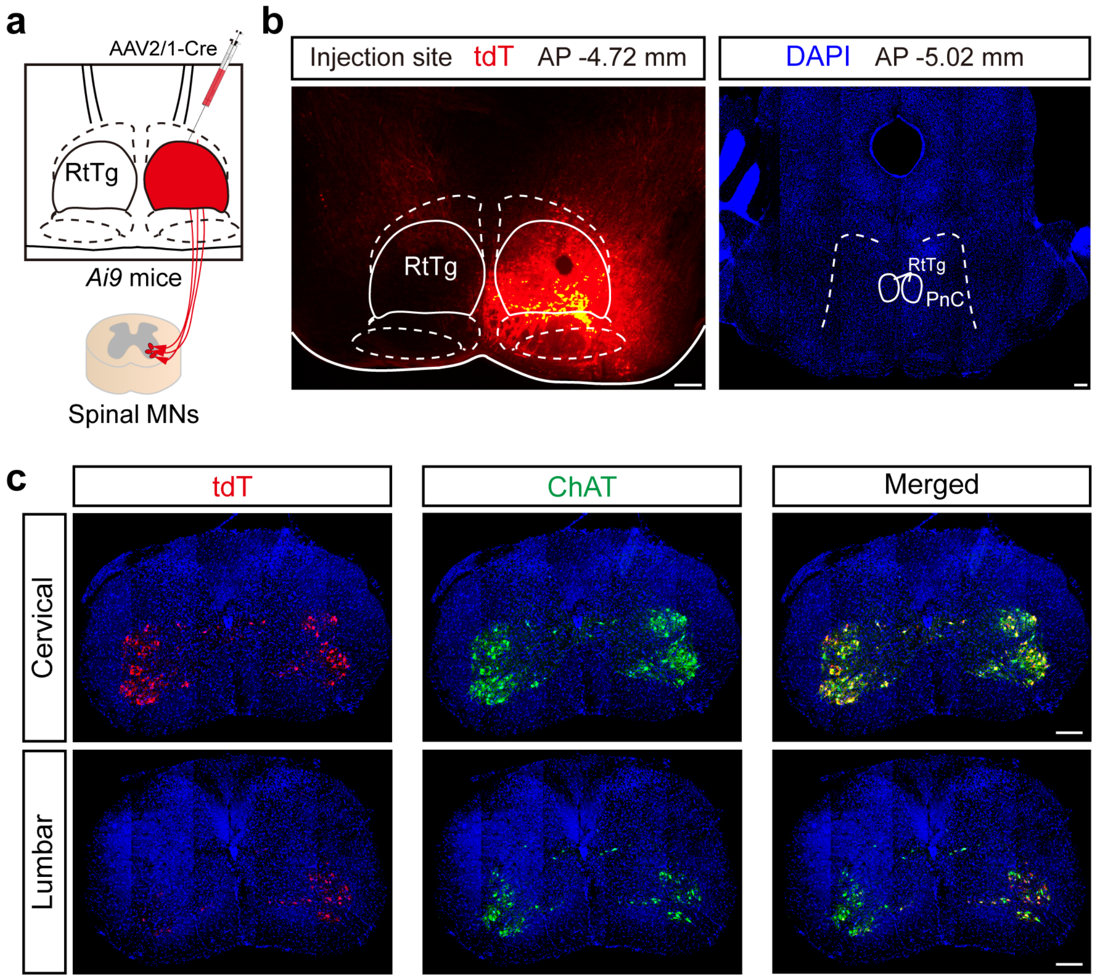

Supplementary Fig. 24. Anterograde transsynaptic tracing from the RtTg to spinal MNs. a Schematic for the AAV2/1-Cre-based anterograde transsynaptic tracing from the RtTg to spinal cord MNs. **b** Representative images showing RtTg injection site (left) and PnC coronal section with no virus cross-contamination. Scale bar, 300 μm. **c** Representative images showing the tdT^+ neuron labelling in coronal sections of spinal cervical and lumbar segments, and the co-labelling of tdT with ChAT. Scale bar, 200 μm.
