# Supplementary material for: A Brainstem reticulotegmental neural ensemble drives acoustic startle reflexes
Source: Nat Commun. 2021 Nov 4;12:6403. doi: 10.1038/s41467-021-26723-9 (PMC8568936; doi:10.1038/s41467-021-26723-9)
Supplement: Supplementary file 2 — Description of Additional Supplementary Files [file 41467_2021_26723_MOESM2_ESM.pdf]

Description of Additional Supplementary Files

Supplementary Movie 1: Optogenetic activation of RtTg $ChR2^{+}$ neurons.

Supplementary Movie 2: Optogenetic activation of RtTg $EGFP^{+}$ neurons.
