# Supplementary material for: A Brainstem reticulotegmental neural ensemble drives acoustic startle reflexes
Source: Nat Commun. 2021 Nov 4;12:6403. doi: 10.1038/s41467-021-26723-9 (PMC8568936; doi:10.1038/s41467-021-26723-9)
Supplement: Supplementary file 5 — Reporting summary [file 41467_2021_26723_MOESM5_ESM.pdf]

nature portfolio

Corresponding author(s): Wei Xiong

Last updated by author(s): Oct 15, 2021

Reporting Summary

Nature Portfolio wishes to improve the reproducibility of the work that we publish. This form provides structure for consistency and transparency in reporting. For further information on Nature Portfolio policies, see our Editorial Policies and the Editorial Policy Checklist.

Statistics

For all statistical analyses, confirm that the following items are present in the figure legend, table legend, main text, or Methods section.

n/a	Confirmed	
[ ]	[x]	The exact sample size (*n*) for each experimental group/condition, given as a discrete number and unit of measurement
[ ]	[x]	A statement on whether measurements were taken from distinct samples or whether the same sample was measured repeatedly
[ ]	[x]	The statistical test(s) used AND whether they are one- or two-sided<br>*Only common tests should be described solely by name; describe more complex techniques in the Methods section.*
[x]	[ ]	A description of all covariates tested
[ ]	[x]	A description of any assumptions or corrections, such as tests of normality and adjustment for multiple comparisons
[ ]	[x]	A full description of the statistical parameters including central tendency (e.g. means) or other basic estimates (e.g. regression coefficient) AND variation (e.g. standard deviation) or associated estimates of uncertainty (e.g. confidence intervals)
[ ]	[x]	For null hypothesis testing, the test statistic (e.g. *F*, *t*, *r*) with confidence intervals, effect sizes, degrees of freedom and *P* value noted<br>*Give P values as exact values whenever suitable.*
[x]	[ ]	For Bayesian analysis, information on the choice of priors and Markov chain Monte Carlo settings
[x]	[ ]	For hierarchical and complex designs, identification of the appropriate level for tests and full reporting of outcomes
[x]	[ ]	Estimates of effect sizes (e.g. Cohen's *d*, Pearson's *r*), indicating how they were calculated

Our web collection on statistics for biologists contains articles on many of the points above.

Software and code

Policy information about availability of computer code

Data collection	For startle reflex tests, startle amplitudes were recorded with Startle Reflex Software (MED Associate). For slice physiological recording, the current and voltage signals were recorded with MultiClamp 700B and Clampex 10 data acquisition software (Molecular Devices, USA). For in vivo electrophysiology recording, the firing rate was recorded with NeuroNexus software (Nex Technologies, USA). For pupil monitoring, pupil size were recorded using an infrared camera-based eye-tracking system (ISCAN systems, USA). For EMG recording and in vivo fiber photometry, we used Spike2 software (CED, Cambridge, UK). Images were captured on a Zeiss LSM880 confocal microscope (Germany).
Data analysis	For gait analysis we used DigiGait software version 12.2 (Mouse Specifics Inc.). For slice physiological recording, the data were analyzed using Clampfit 10.3 (Molecular devices, USA) and MiniAnalysis software version 6.03 (Synaptosoft Inc., USA). Open field behavioral test data were analyzed using ANY-maze software version 5.1 (Global Biotech). For in vivo electrophysiology recording, the data was analyzes using NeuroExplore version 6.0 (Plexon Inc.). For pupil monitoring, EMG recording and in vivo fiber photometry, we used MATLAB 2019a for further analysis. Image J version 1.53a (NIH) were used for cell counting. GraphPad Prism 8 (Graph Pad Software, Inc., USA) were used for the statistical analyses.

For manuscripts utilizing custom algorithms or software that are central to the research but not yet described in published literature, software must be made available to editors and reviewers. We strongly encourage code deposition in a community repository (e.g. GitHub). See the Nature Portfolio guidelines for submitting code & software for further information.

Data

Policy information about availability of data

All manuscripts must include a data availability statement. This statement should provide the following information, where applicable:

- Accession codes, unique identifiers, or web links for publicly available datasets
- A description of any restrictions on data availability
- For clinical datasets or third party data, please ensure that the statement adheres to our policy

The in situ hybridization database from Allen Mouse Brain Atlas (https://mouse.brain-map.org/experiment/show/73818754) (https://mouse.brain-map.org/experiment/show/72081554) were used. The data that support the findings of this study are provided in the article and its Supplementary information files, and are available from the corresponding author upon reasonable request. Source data are provided with this paper.

Field-specific reporting

Please select the one below that is the best fit for your research. If you are not sure, read the appropriate sections before making your selection.

[x] Life sciences   [ ] Behavioural & social sciences   [ ] Ecological, evolutionary & environmental sciences

For a reference copy of the document with all sections, see nature.com/documents/nr-reporting-summary-flat.pdf

Life sciences study design

All studies must disclose on these points even when the disclosure is negative.

Sample size	No statistical methods were used to pre-determine sample sizes but our sample sizes are similar to those reported in previous publications (PMID: 32209479; PMID: 29779945; PMID: 24390226).
Data exclusions	For all experiments, mice with signs of infection/bleeding/unhealthy conditions after the surgeries were excluded for behavioral tests and mice with missed viral injections or implantation targets, as described by brain atlas, were not included in experimental analyses.
Replication	Behavioral experiments are replicated multiples times with independent mice, and at least two people independently analyzed time points for the behavioral events. Numbers of replicates (n) are indicated in the figure legends.
Randomization	The animals in the behavioral tests were randomized assigned.
Blinding	All investigators were blinded to group allocation during data collection and analysis.

Reporting for specific materials, systems and methods

We require information from authors about some types of materials, experimental systems and methods used in many studies. Here, indicate whether each material, system or method listed is relevant to your study. If you are not sure if a list item applies to your research, read the appropriate section before selecting a response.

Materials & experimental systems

n/a	Involved in the study	
[ ]	[x]	Antibodies
[x]	[ ]	Eukaryotic cell lines
[x]	[ ]	Palaeontology and archaeology
[ ]	[x]	Animals and other organisms
[x]	[ ]	Human research participants
[x]	[ ]	Clinical data
[x]	[ ]	Dual use research of concern

Methods

n/a	Involved in the study	
[x]	[ ]	ChIP-seq
[x]	[ ]	Flow cytometry
[x]	[ ]	MRI-based neuroimaging

Antibodies

Antibodies used	Primary antibodies used in the present study are as follows:<br>Rabbit Polyclonal anti-c-fos; Abcam, Cat#ab190289; dilution 1:1000<br>Mouse Monoclonal anti-NeuN, clone A60; Millipore, Cat#MAB377; dilution 1:500<br>Goat Polyclonal anti-ChAT; Millipore, Cat#MAB144P; dilution 1:200<br>Rabbit Polyclonal anti-glutamate; Sigma, Cat#G6642; dilution 1:500<br>Rabbit Polyclonal anti-GFP; Thermo Fisher, Cat#A-11122; dilution 1:100<br>Secondary antibodies used in the present study are as follows:

	Goat anti-rabbit Alexa 488-conjugated secondary antibody; CST, Cat#4412S; dilution 1:1000<br>Goat anti-rabbit Alexa 594-conjugated secondary antibody; CST, Cat#8889S; dilution 1:1000<br>Goat anti-mouse Alexa 594-conjugated secondary antibody; Thermo Fisher, Cat#A-11032; dilution 1:1000<br>Dunkey anti-goat Alexa 488-conjugated secondary antibody; Thermo Fisher, Cat#A-11055; dilution 1:200
Validation	anti-c-fos (e.g. PMID: 33431851)<br>anti-NeuN (e.g. PMID: 34400844)<br>anti-glutamate (e.g. PMID: 7020652)<br>anti-ChAT (e.g. PMID: 29084947)<br>anti-GFP (e.g. PMID: 33941892)<br>Goat anti-rabbit Alexa 488-conjugated secondary antibody (e.g. PMID: 33762736)<br>Goat anti-rabbit Alexa 594-conjugated secondary antibody (e.g. PMID: 34234149)<br>Goat anti-mouse Alexa 594-conjugated secondary antibody (e.g. PMID: 30718913)<br>Dunkey anti-goat Alexa 488-conjugated secondary antibody (e.g. PMID: 34497373)

Animals and other organisms

Policy information about studies involving animals; ARRIVE guidelines recommended for reporting animal research

Laboratory animals	We used C57BL/6J, Vglut2-Cre, Vgat-Cre and GAD1-EGFP mice male mice aged 8-10 weeks. C57BL/6J mice (#213) purchased from Beijing Vital River; Vglut2-Cre (#016963), Vgat-Cre (#016962), GAD1-EGFP(#007677) male mice purchased from Jackson Laboratories. The mice were group-housed with 3-5 per cage in a colony in a stable environment (23-25 ºC ambient temperature and 50% humidity) unless a cannula or optical fiber was implanted. They were maintained under a 12-hour light/dark cycle (lights on from 7:00 a.m. to 7:00 p.m.) with water and food available ad libitum.
Wild animals	The study did not involve wild animals.
Field-collected samples	This study did not involve samples collected from the field.
Ethics oversight	All animal protocols were approved by the Animal Care and Use Committee of the University of Science and Technology of China.

Note that full information on the approval of the study protocol must also be provided in the manuscript.
